# Regulation of the pyrimidine biosynthetic pathway by lysine acetylation of *E. coli* OPRTase

**DOI:** 10.1111/febs.16598

**Published:** 2022-09-02

**Authors:** Gema Lozano‐Terol, Julia Gallego‐Jara, Rosa Alba Sola‐Martínez, Álvaro Ortega, Adrián Martínez Vivancos, Manuel Cánovas Díaz, Teresa de Diego Puente

**Affiliations:** ^1^ Department of Biochemistry and Molecular Biology and Immunology (B), Faculty of Chemistry University of Murcia Spain

**Keywords:** *E. coli*, lysine acetylation, OPRTase, pyrimidine biosynthesis pathway, regulation

## Abstract

The *de novo* pyrimidine biosynthesis pathway is an important route due to the relevance of its products, its implications in health and its conservation among organisms. Here, we investigated the regulation by lysine acetylation of this pathway. To this aim, intracellular and extracellular metabolites of the route were quantified, revealing a possible blockage of the pathway by acetylation of the OPRTase enzyme (orotate phosphoribosyltransferase). Chemical acetylation of OPRTase by acetyl‐P involved a decrease in enzymatic activity. To test the effect of acetylation in this enzyme, K26 and K103 residues were selected to generate site‐specific acetylated proteins. Several differences were observed in kinetic parameters, emphasizing that the *k*_cat_ of these mutants showed a strong decrease of 300 and 150‐fold for OPRTase‐103AcK and 19 and 6.3‐fold for OPRTase‐26AcK, for forward and reverse reactions. *In vivo* studies suggested acetylation of this enzyme by a nonenzymatic acetyl‐P‐dependent mechanism and a reversion of this process by the CobB deacetylase. A complementation assay of a deficient strain in the *pyrE* gene with OPRTase‐26AcK and OPRTase‐103AcK was performed, and curli formation, stoichiometric parameters and orotate excretion were measured. Complementation with acetylated enzymes entailed a profile very similar to that of the ∆*pyrE* strain, especially in the case of complementation with OPRTase‐103AcK. These results suggest regulation of the *de novo* pyrimidine biosynthesis pathway by lysine acetylation of OPRTase in *Escherichia coli*. This finding is of great relevance due to the essential role of this route and the OPRTase enzyme as a target for antimicrobial, antiviral and cancer treatments.

## Graphical abstract

The *de novo* pyrimidine biosynthesis pathway is an important route due to the relevance of its products and implications in health. Here, we found that lysine acetylation of the OPRTase enzyme at K26 and K103 involved a blockage in pyrimidine biosynthesis and, consequently, an accumulation of orotate and a strong decrease in cell growth. These results suggest regulation of this pathway by lysine acetylation of OPRTase in *Escherichia coli*.

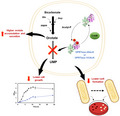

**Abbreviations**
OMP: orotidine 5′‐monophosphate
OPRTase: orotate phosphoribosyltransferase
PRPP: 5‐phospho‐α‐d‐ribose 1‐diphosphate
UMP: uridine 5‐monophosphate
wt: wild‐type

## Introduction

**Fig. 1:**
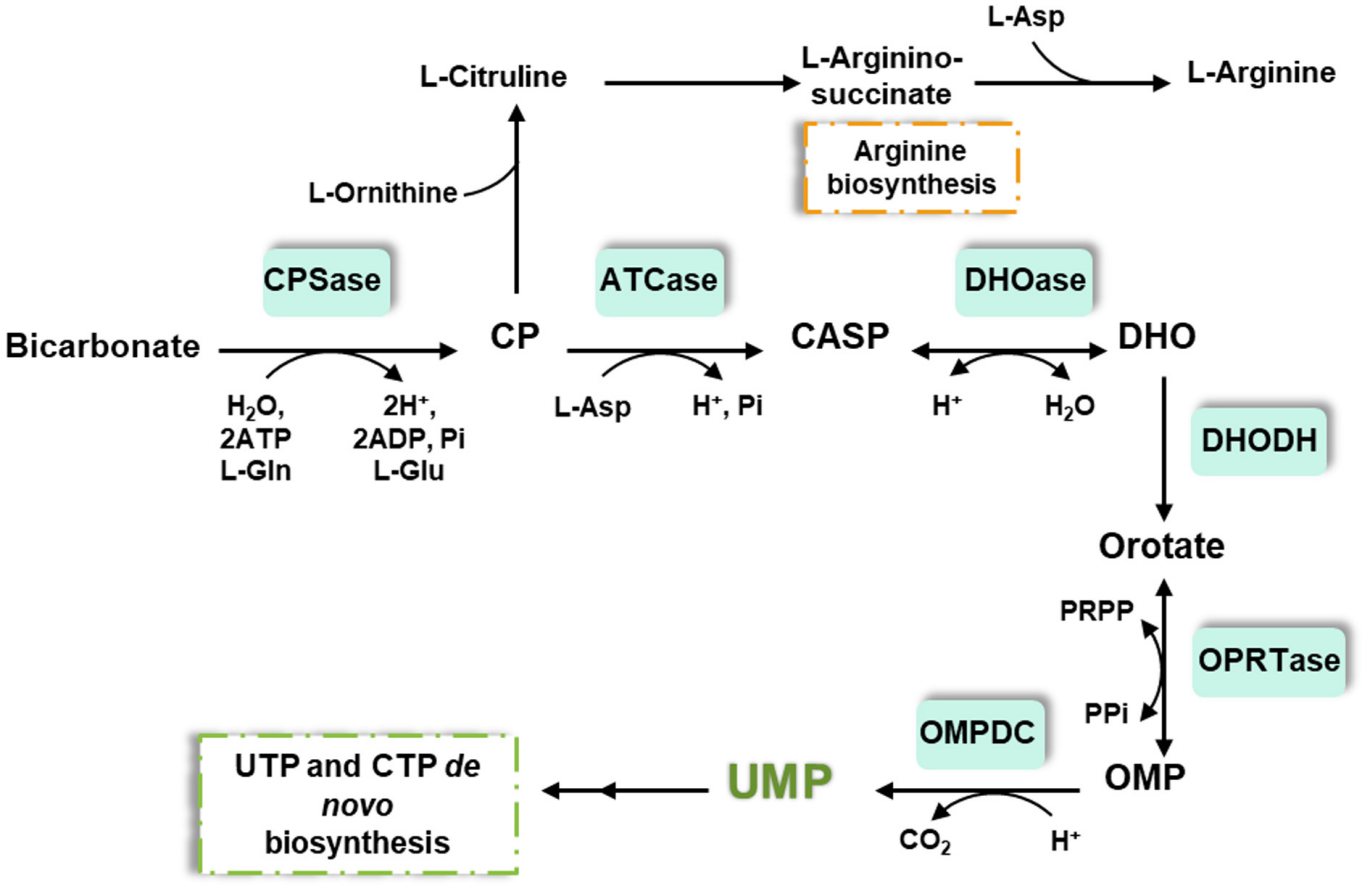
*Escherichia coli de novo* pyrimidine biosynthesis pathway. CPSase, carbamoyl phosphate synthetase enzyme; CP, carbamoyl phosphate; CASP, *N*‐carbamoyl‐l‐aspartate; ATCase, aspartate carbamoyltransferase; DHOase, dihydroorotase; DHO, dihydroorotate; DHODH, dihydroorotate dehydrogenase; OPRTase, orotate phosphoribosyltransferase; OMP, orotidine 5′‐monophosphate; OMPDC, orotidine 5′‐phosphate decarboxylase; UMP, uridine 5′‐monophosphate.

Pyrimidine and purine nucleotides are essential compounds in all living organisms since they are the building blocks of nucleic acids. Nucleotides are also required for phospholipids and glycogen biosynthesis and are involved in intermediary metabolism as constituents of coenzymes, phosphorylation reagents, metabolic signal molecules and allosteric modulators [1, 2]. Purine and pyrimidine nucleotides are synthesized *de novo* or reutilized from exogenous free bases and nucleosides in the salvage pathways. The *de novo* pyrimidine nucleotide biosynthetic pathway is conserved among organisms to produce uridine 5‐monophosphate (UMP), the precursor of all pyrimidine nucleotides which is subsequently transformed into UTP and CTP [1, 3, 4]. In *Escherichia coli* bicarbonate is converted to UMP in six steps, involving l‐aspartate, l‐glutamine and 5‐phospho‐α‐d‐ribose 1‐diphosphate (PRPP). The first step, carried out by carbamoyl phosphate synthetase enzyme (CPSase or CarAB; EC 6.3.5.5), is common for pyrimidines and arginine biosynthesis pathways. CPSase synthetizes carbamoyl phosphate (CP) from bicarbonate, ATP and l‐glutamine [5]. The CP is condensed with l‐aspartate to form *N*‐carbamoyl‐l‐aspartate (CASP) by the aspartate carbamoyltransferase (ATCase or PyrBI; EC 2.1.3.2) [6]. The third step, performed by the dihydroorotase (DHOase or PyrC; EC 3.5.2.3), is the cyclization of the CASP to form the dihydroorotate (DHO), the first pyrimidine ring compound of the route [7]. Then, DHO is oxidated to orotate by the action of the dihydroorotate dehydrogenase (DHODH or PyrD; EC 1.3.5.2) [8]. The fifth step is carried out by the orotate phosphoribosyltransferase (OPRTase or PyrE; EC 2.4.2.10) and consists of the formation of the first pyrimidine nucleotide, the orotidine 5′‐monophosphate (OMP), by the condensation of orotate and PRPP [9]. Finally, OMP is decarboxylated to UMP by the orotidine 5′‐phosphate decarboxylase (OMPDC or PyrF; EC 4.1.1.23) [10] (Fig. 1).

The *de novo* pyrimidine biosynthetic pathway is regulated at the gene expression level and by allosteric enzymes to conserve resources because of the relevance of the products of this route and its implications in health [2]. In humans, cellular pyrimidine deficiencies trigger innate immune signalling [11] and the mutation or deficiency of some genes of the pathway leads to serious metabolic disorders, such as hereditary orotic aciduria [12] and neurological disorders [13], such as Miller syndrome [14]. Interestingly, an unbalance in this pathway has been found in the brain of patients with Alzheimer's disease, therefore uridine supplementation might be used as a treatment for those patients [15]. Furthermore, the upregulation of some enzymes in this pathway indicates a poor prognosis in cancer patients, hence these genes are attractive anticancer drugs to inhibit proliferation and metastasis [16, 17, 18, 19]. Likewise, viral replication relies on the host to supply nucleotides, thus the enzymes in this pathway are targets for antiviral treatments [20, 21, 22, 23]. Moreover, the inhibition of pyrimidine biosynthesis enzymes is employed for the treatment of microorganism and parasite infections [24, 25, 26, 27].

In this work, we focus on OPRTase, the fifth enzyme of the *de novo* biosynthetic route, which belongs to the phosphoribosyltransferase (PRTases) family. PRTases enzymes play a key role in the metabolism of pyrimidine and purine nucleotides and in the biosynthesis of tryptophan and histidine [28]. PRTases constitute two evolutionary groups based on different active site architectures. Type I enzymes, among which are the OPRTases, show a Rossman fold and a solvent‐exposed active site architecture, whereas type II enzyme's active site constitutes a TIM barrel architecture [29]. This last group includes the nicotinamide phosphoribosyltransferase (NAMPT) whose activity is regulated by acetylation [30]. In humans, PRTases enzymes have been largely studied and changes in the DNA sequence of their genes are associated with pathologies such as 2,8‐dihydroxyadenine lithiasis, Lesch–Nyhan syndrome and orotic aciduria [31]. Furthermore, the OPRTase enzyme contributes to phosphorylation and activation of the chemotherapy prodrug 5‐fluorouracil [32]. Likewise, this enzyme constitutes an attractive target for antiparasitic or antimicrobial treatments [24, 25]. OPRTase is encoded by the *pyrE* gene and its expression is regulated by transcriptional attenuation due to the UTP pool [2, 33, 34, 35]. It is important to point out that the acetylation of the OPRTase has been noted by several studies in *E. coli* [36, 37, 38, 39, 40, 41], but the impact of acetylation on this enzyme is unknown.

*N*ε‐lysine acetylation is an important posttranslational protein modification (PTM) that controls many cell processes [42]. In *E. coli* the most studied acetyltransferase (KAT) is YfiQ [43], also known as Pka and PatZ, although other acetyltransferase enzymes, such as YiaC, have been identified [37]. Nevertheless, accumulating evidence indicates that the main acetylation pathway in this bacterium is the nonenzymatic acetyl‐P dependent pathway, and global acetylation is positively correlated with an intracellular acetyl‐P concentration in response to glucose consumption and nitrogen starvation [36, 38, 44]. Furthermore, lysine acetylation, from the enzymatic or nonenzymatic mechanism, can be reverted by the only known NAD^+^‐dependent (Sir2‐like) deacetylase in *E. coli*, called CobB [44, 45, 46]. However, CobB‐dependent deacetylation acts on only 10% of the detected acetylated lysines [40, 46].

The present study aims to unveil if the *de novo* pyrimidine biosynthesis pathway could be regulated by lysine acetylation in *E. coli*. To achieve this objective intracellular and extracellular concentrations of the metabolites of the route were measured in the strain deficient in CobB deacetylase and in the wild‐type (wt) strain. Results showed significant differences in orotate and UMP concentrations between both strains, which suggested regulation of the route by lysine acetylation. Lower activity was observed for OPRTase acetylated *in vitro*, and two lysine residues, K26 and K103, were selected and modified by the genetic code expansion strategy. The acetylation of K26 and K103 residues of OPRTase involved a great loss of activity *in vitro*. Moreover, *in vivo* complementation assays of a strain deficient in *pyrE* gene supplemented with OPRTase with K26 or K103 residues acetylated showed no recovery of the OPRTase functions, with similar behaviour to the Δ*pyrE* strain. These results suggest regulation of *de novo* pyrimidine biosynthesis pathway by lysine acetylation of OPRTase, an essential enzyme of the *de novo* pyrimidine route and an attractive target for antimicrobial, antiviral and anticancer treatments.

## Results

### ∆ *cobB* and wt *E. coli* strains show differences in pyrimidine biosynthesis metabolites

**Fig. 2:**
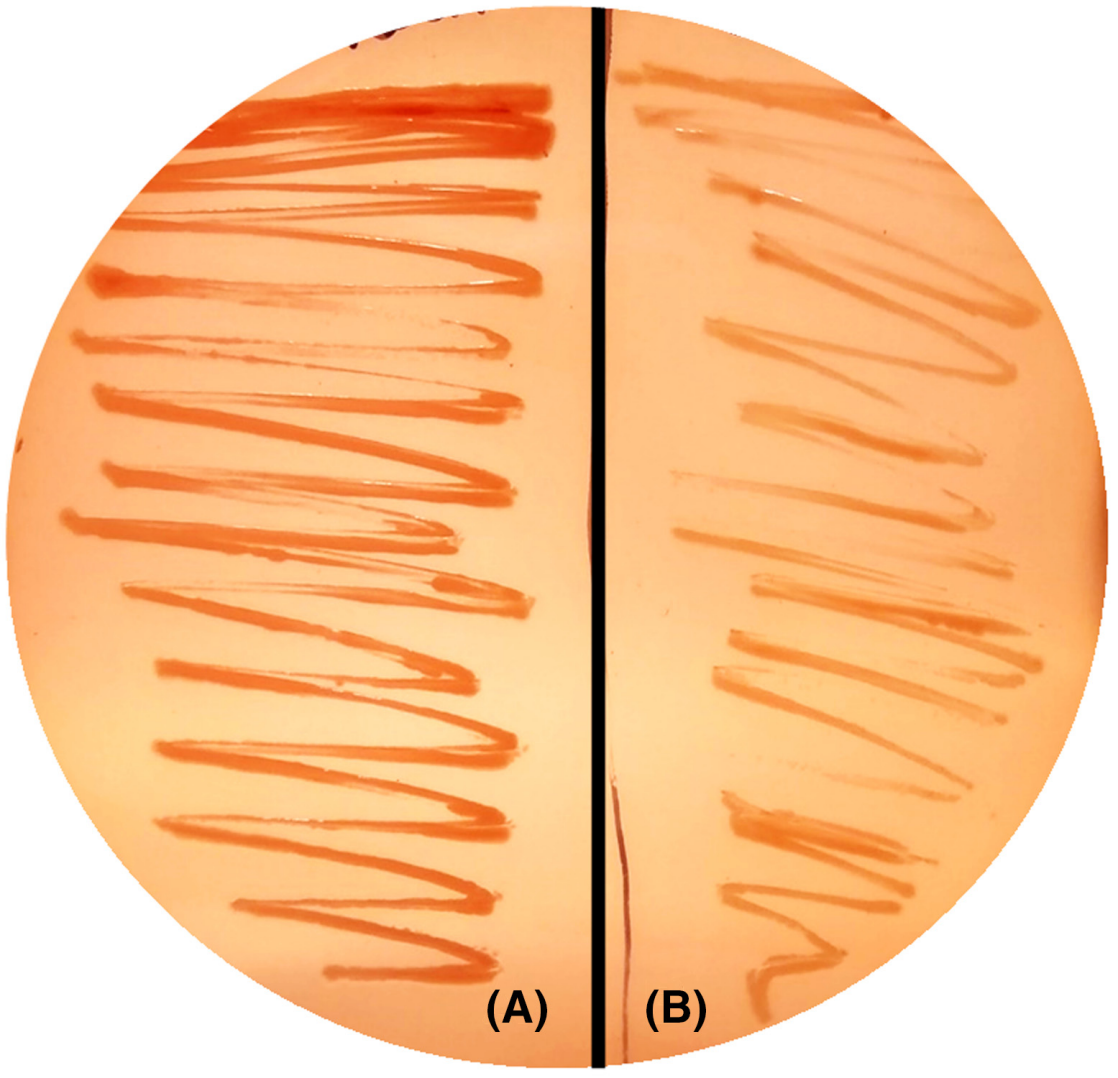
Congo red binding assay of *Escherichia coli* wt (A) and Δ*cobB* (B) strains. Both strains were spotted in CR medium and grown at 30 °C for 20 h, and dye binding was detected after incubation at 4 °C for 48 h. Image was analysed by open‐source software imagej (National Institutes of Health, Bethesda, MA, USA), and nine regularly spaced points were selected in each part of the plate, A and B, with Multi‐Point Tool. Average intensity of each multi‐point set was 897.7 ± 44 for A and 345.1 ± 43 for B, resulting in a 38.5% reduction in intensity for *cobB* mutant compared with wt.

Orotate and other intermediary metabolites of the pyrimidine biosynthesis pathway are excreted during the growth of some *E. coli* strains [47, 48, 49]. In our previous work, we found a 35‐fold increase in orotate excretion in the strain deficient in the gene of the deacetylase *cobB* with respect to the wt strain [50]. In that study, we assumed that the higher orotate excretion in Δ*cobB* strain could be due to the control of the α‐ketoglutarate node by acetylation of isocitrate lyase. Nevertheless, this great difference probably cannot be only explained by this fact, and simultaneously, a regulation by acetylation of the pyrimidine biosynthesis pathway in the absence of CobB deacetylase could happen, which would contribute to the high increase in orotate excretion. Consistent with this proposal, we analysed curli fibres production by a Congo red binding assay in the wt strain and the *cobB* mutant as an indirect measurement of the perturbation of UMP biosynthesis. Garavaglia et al. [51] demonstrated that the inactivation of the pyrimidine biosynthesis pathway impairs curli and cellulose production in *E. coli*. Curli amyloid fibres bind to Congo red dye and show a red phenotype in agar medium supplemented with this dye (CR medium). As a result of these assays, wt strain displayed a darker CR medium phenotype than ∆*cobB* strain (Fig. 2), which could indicate altered curly formation because of lower production of pyrimidines in this mutant.

**Fig. 3:**
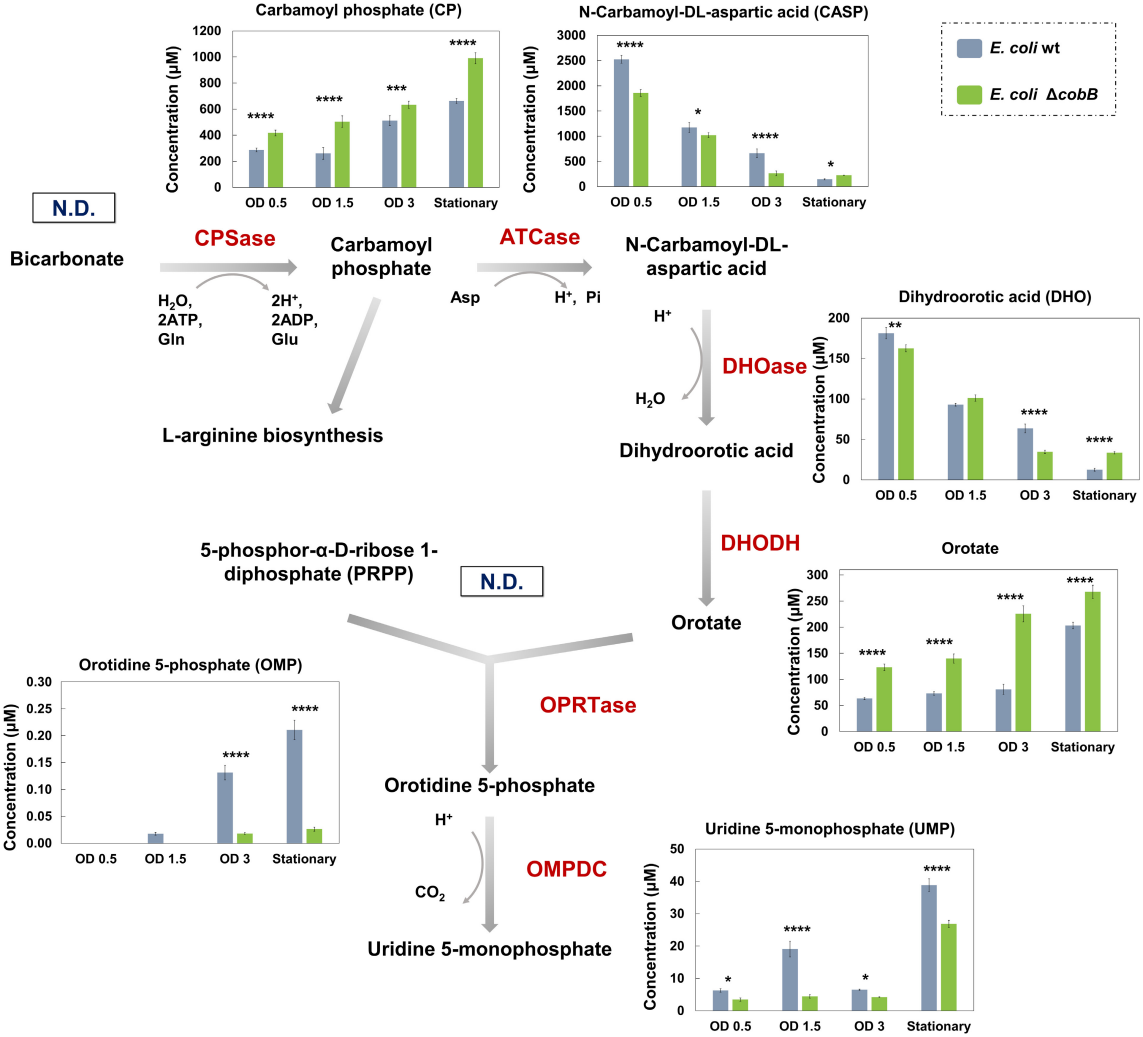
Intracellular concentration of the metabolites of the pyrimidine biosynthesis pathway. *Escherichia coli* K12 wt (Grey bars) and Δ*cobB* (green bars) strains were grown in minimal M9 medium supplemented with glucose 20 mm. N.D., not detected. OD refers to OD_600_. *De novo* pyrimidine biosynthesis pathway with the charts of the intracellular concentration of CP, CASP, DHO, orotate, OMP and UMP in both strains at different times of growth. A two‐way ANOVA test was carried out with graphpad prism 7.0 to identify differences in concentrations between both strains [*P*‐value < 0.0001 (****), < 0.001 (***), < 0.01 (**) and < 0.05 (*)]. Error bars are standard errors calculated from two repeats.

**Fig. 4:**
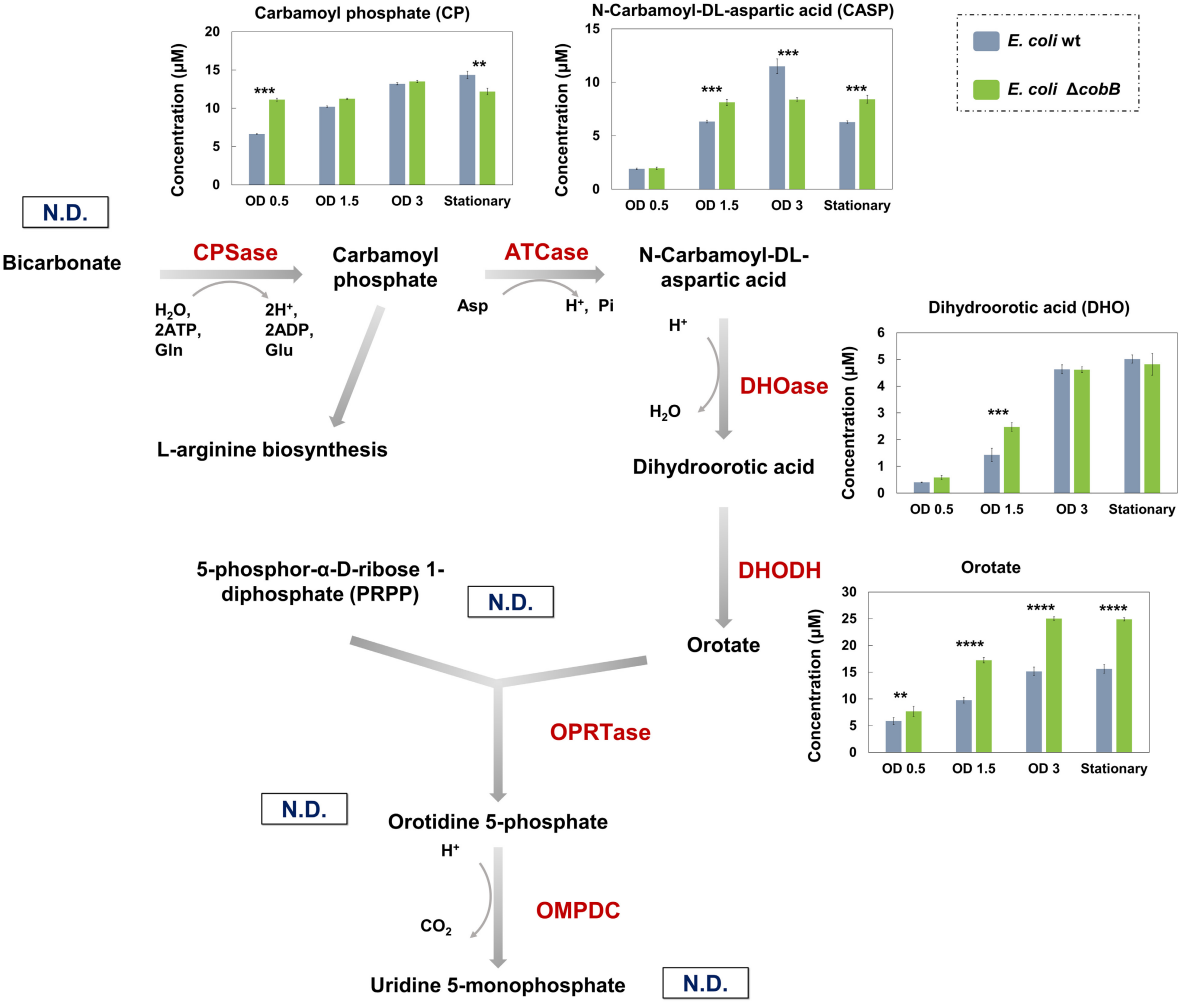
Extracellular concentration of the metabolites of the pyrimidine biosynthesis pathway. *Escherichia coli* K12 wt (Grey bars) and Δ*cobB* (green bars) strains were grown in minimal M9 medium supplemented with glucose 20 mm. N.D, not detected. OD refers to OD_600_. *De novo* pyrimidine biosynthesis pathway with the charts of the extracellular concentration of CP, CASP, DHO and orotate in both strains at different times of growth. A two‐way ANOVA test was carried out with graphpad prism 7.0 to identify differences in concentrations between both strains [*P*‐value < 0.0001 (****), < 0.001 (***), < 0.01 (**) and < 0.05 (*)]. Error bars are standard errors calculated from two repeats.

**Fig. 5:**
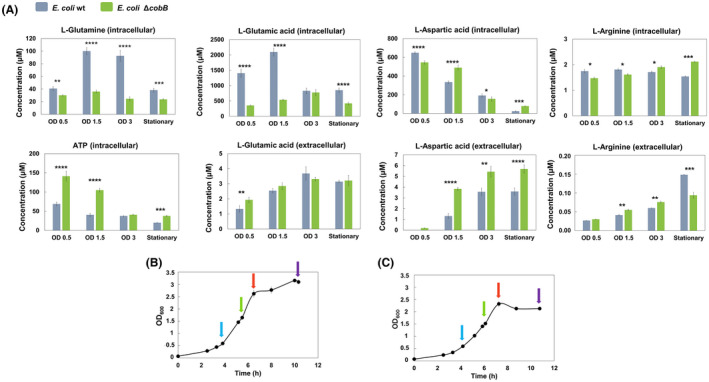
Intracellular and extracellular concentration of other metabolites involved in the reactions of the pyrimidine biosynthesis pathway. *Escherichia coli* K12 wt (Grey bars) and ΔcobB (green bars) strains were grown in minimal M9 medium supplemented with glucose 20 mm. N.D, not detected. OD refers to OD_600_. (A) Charts with the intracellular and extracellular concentration of other metabolites that are involved in the route (Glu, Gln, Asp, Ar and ATP) in both strains and at different times of growth. A two‐way ANOVA test was carried out with graphpad prism 7.0 to identify differences in concentrations between both strains [*P*‐value < 0.0001 (****), < 0.001 (***), < 0.01 (**) and < 0.05 (*)]. *E. coli* K12 wt (B) and *E. coli* K12 ΔcobB (C) strains grown at OD_600_ with arrows indicating the culture time at which the samples were taken for the quantification of intracellular and extracellular metabolites: Before exponential growth phase (OD_600_ 0.5) (blue), in mid‐exponential growth phase (OD_600_ 1.5) (green), in long‐exponential growth phase (OD_600_ 3) (red) and in stationary growth phase (stationary) (purple). Error bars are standard errors calculated from two repeats.

In view of these promising results, we measured the intracellular and extracellular concentration of the metabolites of the *de novo* pyrimidines biosynthesis pathway by LC–MS/MS in *E. coli* wt and *E. coli* Δ*cobB* strains growing in minimal medium supplemented with glucose. Samples were taken at different times of growth: before exponential growth phase (OD_600_ 0.5), in mid‐exponential growth phase (OD_600_ 1.5), in long‐exponential growth phase (OD_600_ 3) and in stationary growth phase (Stationary) (Fig. 5B,C). The main intermediary metabolites of the route and other metabolites involved in the reactions (bicarbonate, CP, CASP, DHO, orotate, PRPP, OMP, UMP, l‐glutamine, l‐glutamate, ATP and l‐aspartic acid) were analysed (Figs 3, 4, 5).

Production or consumption profiles of the main metabolites of UMP synthesis are shown in Fig. 3. Bicarbonate and PRPP were not detected under the conditions of the study. We observed a significantly higher concentration of CP in Δ*cobB* strain than in wt strain in all samples (*P*‐value < 0.001). The following two metabolites of the pathway, CASP and DHO, showed a similar trend in both strains, with a decrease in concentration along culture time. Regarding orotate, a significantly greater concentration in Δ*cobB* than in wt strain (*P*‐value < 0.0001) was detected, with an increase in its values from 123.1 to 267.9 μm in Δ*cobB* strain and from 63.3 to 203.3 μm in wt strain. The next metabolite of the pathway OMP was not detected in the early stages of the culture growth for both strains; however, it was detected in the late phase of cultivation, with higher abundance in the wt strain. The last metabolite of the route, the UMP, showed higher values in wt than in Δ*cobB* strain in all samples (*P*‐values < 0.05 and < 0.0001), ranging from 6.3 to 38.8 μm in wt and from 3.4 to 26.8 μm in Δ*cobB* strain. In summary, the orotate and OMP concentration profiles suggested a different activity of the OPRTase enzyme in both strains, resulting in a lower final UMP concentration for the Δ*cobB* strain.

As regards extracellular metabolites shown in Fig. 4, we observed no excretion of OMP or UMP. Furthermore, all the quantified metabolites showed the same profile with increasing concentration during growth (Fig. 4). The greatest differences between strains were observed in CASP and orotate. CASP showed a lower concentration in Δ*cobB* strain than in wt strain in OD_600_ 1.5 and stationary samples (*P*‐values < 0.001), and the opposite behaviour in OD_600_ 3 sample (*P*‐values < 0.001). Lastly, the orotate was the metabolite with the greatest differences in extracellular concentration between both strains, with a higher concentration in Δ*cobB* strain (*P*‐value < 0.0001), which corroborates a blockage of the metabolic pathway at the level of the OPRTase enzyme in the *cobB* mutant.

Concerning the concentration of the other metabolites, ATP, glutamine and glutamate, which take part in the first reaction of the route [5], showed a different profile (Fig. 5A). ATP was not excreted to the extracellular medium; however, ATP intracellular concentration was significantly higher in Δ*cobB* than in wt strain and decreased during the culture time. Regarding glutamate and glutamine intracellular concentrations, higher values in wt than in Δ*cobB* strain were detected (*P*‐value < 0.01 and < 0.0001). Glutamine was not excreted to the extracellular medium, but glutamate was excreted with almost no difference between both strains. CP, the first product of the pathway is also involved in arginine biosynthesis [5], so we measured the intracellular and extracellular arginine concentration, but we did not observe relevant differences between both strains. Finally, aspartate takes part in ATCase reaction [6] and showed higher extracellular concentration in Δ*cobB* than in wt strain (*P*‐value < 0.01 and < 0.0001).

Considering these results, while there are other differences between wt and ∆*cobB*, the major difference is the drop in OMP and the orotate build‐up in Δ*cobB*, which suggests a blockage at the level of the OPRTase enzyme in this mutant that could be due to an acetylation event. Another appreciable difference was a significantly higher concentration of CP and aspartate in Δ*cobB* compared with wt strain, which could also point to different regulation of the ATCase enzyme in both strains.

### Lysine acetylation regulates the OPRTase activity

**Fig. 6:**
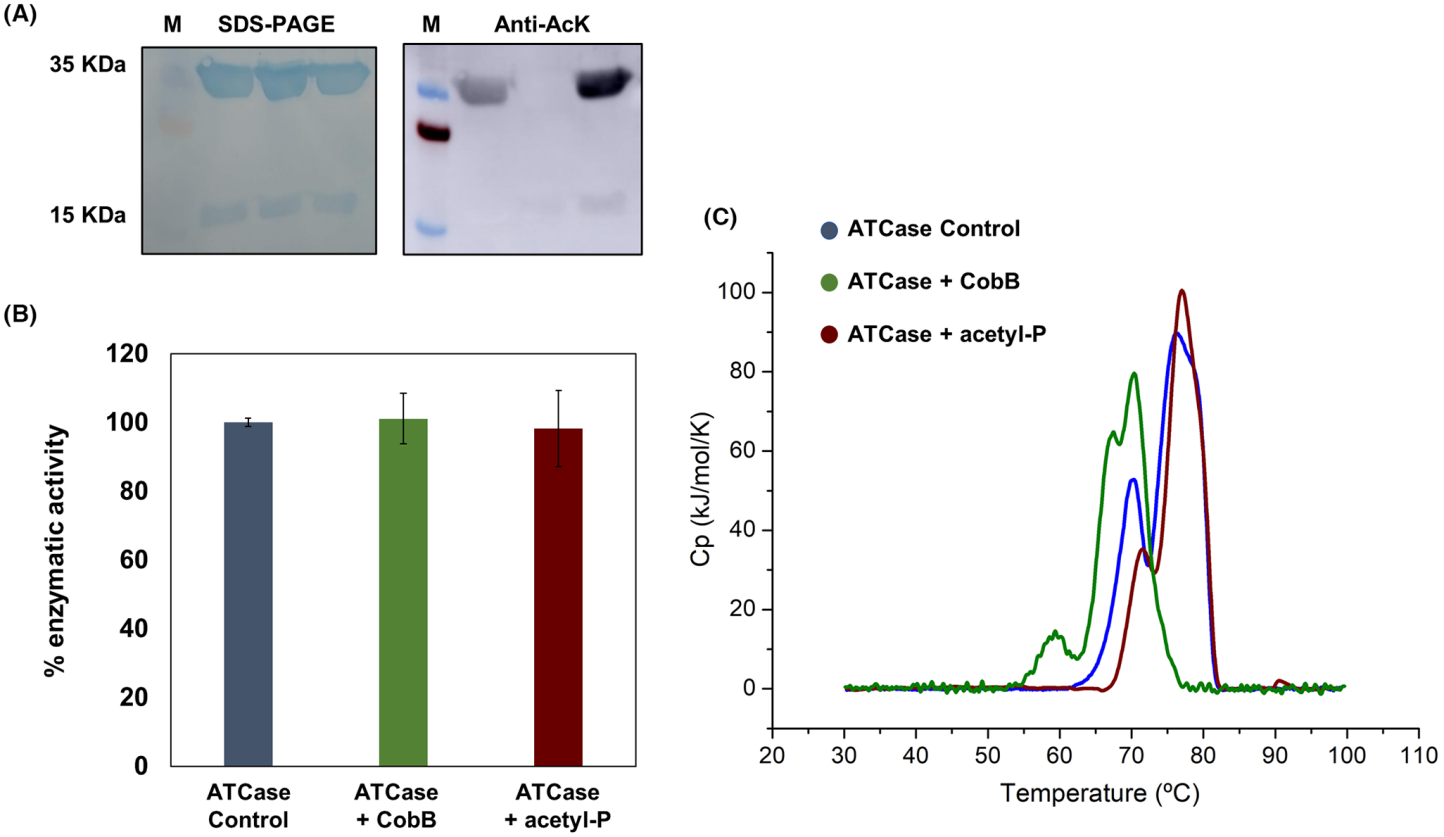
Analysis of acetylation, enzymatic activity and thermostability of ATCase control, ATCase subjected to a deacetylation reaction (ATCase + CobB) by adding CobB and ATCase subjected to an acetylation reaction with acetyl‐P 10 mm (ATCase + acetyl‐P). (A) SDS/PAGE and anti‐acetyl‐lysine (anti‐AcK) western blot of ATCase control (lane 1), ATCase + CobB (lane 2) and ATCase + acetyl‐P (lane 3) samples. The molecular mass marker (M) is included in the right side. (B) Measurement of the enzyme activity of ATCase control, ATCase + CobB and ATCase + acetyl‐P taking as 100% the value of ATCase control. (C) DSC analysis of ATCase control, ATCase + CobB and ATCase + acetyl‐P samples. Error bars are standard errors calculated from three repeats. For these assays, proteins were purified from cultures of *Escherichia coli* BL21 (DE3) transformed with pRSET‐*pyrBI* vector and grown in LB medium.

To verify whether lysine acetylation involved changes in ATCase or OPRTase activity, we purified both enzymes in an LB medium and subjected them to a chemical acetylation process using 10 mm acetyl‐P and to a deacetylation reaction with CobB. Although carbamoyl phosphate build‐up could suggest ATCase activity is altered, no differences in activity measurements were observed between purified, acetylated and deacetylated enzymes. Instead, the intense signal was detected on the bands of ATCase control and ATCase acetylated, while the deacetylated enzyme showed no signal by western blot with anti‐AcK antibody. Then, we perform thermostability tests by DSC, which showed a decrease in the Tm values for the two peaks, around 5–10 °C, in the deacetylated sample with respect to the acetylated and control samples (Fig. 6). The denaturation events of this enzyme have been reported in previous studies [52]. Therefore, the acetylation of ATCase does not seem to affect the enzymatic activity, but it could promote the thermal stability of the enzyme.

**Fig. 7:**
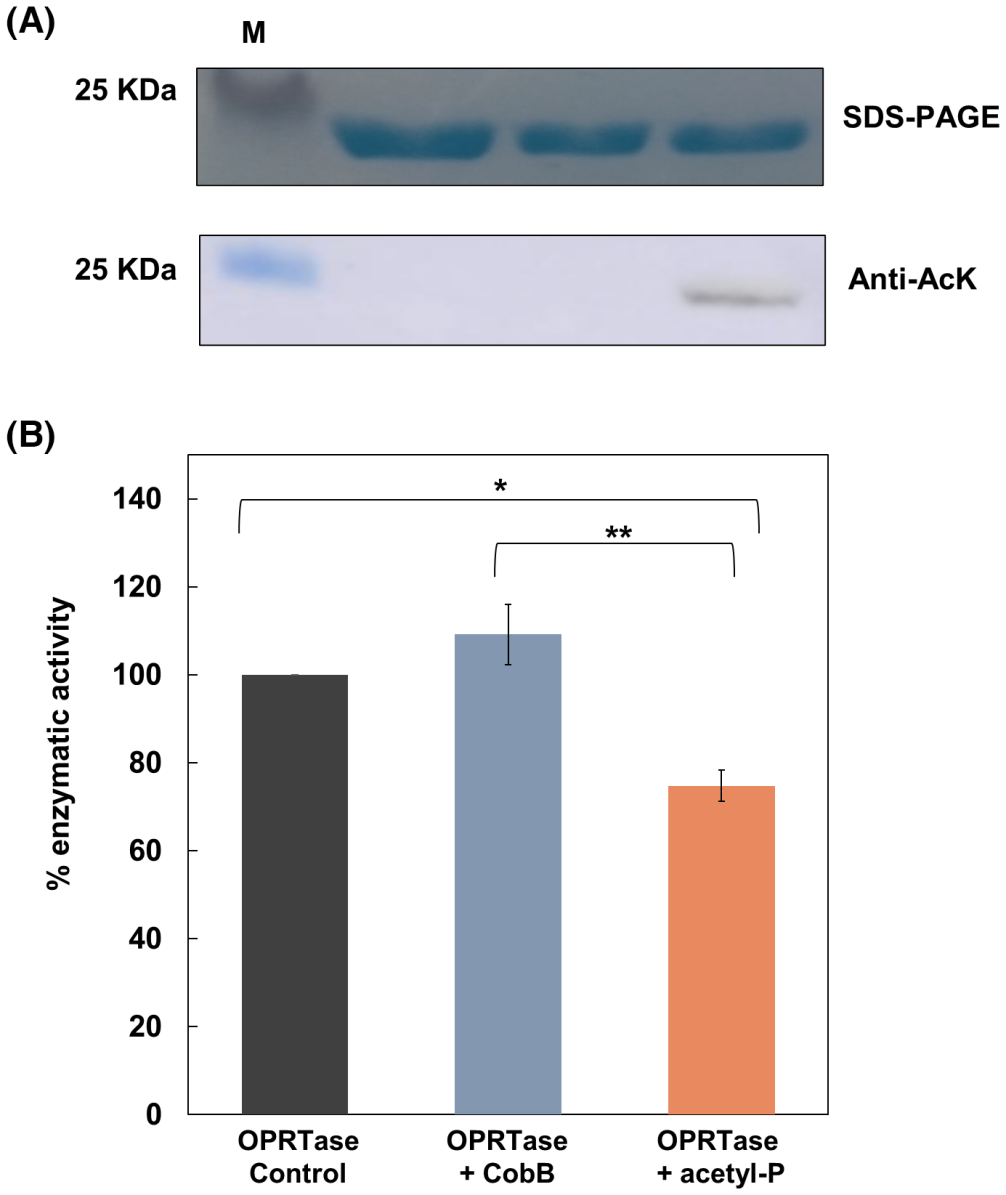
Western blot and enzymatic activity of OPRTase purified and subjected to control reaction (OPRTase control), OPRTase subjected to a deacetylation reaction (OPRTase + CobB) by adding CobB and OPRTase subjected to an acetylation reaction with acetyl‐P 10 mm (OPRTase + acetyl‐P). (A) SDS/PAGE and western blot anti‐AcK analysis of OPRTase control (lane 1), OPRTase + CobB (lane 2) and OPRTase + acetyl‐P (lane 3) samples. The molecular mass marker (M) is included in the left side. (B) Relative enzymatic activity of OPRTase control, OPRTase + CobB and OPRTase + acetyl‐P. The activity of OPRTase control sample was set to 100%. A one‐way ANOVA test was carried out to identify significant differences between relative enzymatic activities [*P*‐value < 0.01 (**) and < 0.05 (*)]. Error bars are standard errors calculated from three repeats. For these assays, proteins were purified from cultures of *Escherichia coli* BL21 (DE3) transformed with pRSET‐*pyrE* vector and grown in LB medium.

Regarding OPRTase, to check its acetylation, we performed a western blot using the anti‐AcK antibody (Fig. 7A). We observed no signal in control and deacetylated samples and a slight increase in the signal of acetylated sample. Then, we measured the forward OPRTase catalysed reaction with PRPP and orotate as substrates. A decrease of 25.2% in acetylated OPRTase enzymatic activity with respect to OPRTase control (*P*‐value < 0.05) was observed. This loss of activity was higher when comparing deacetylated and acetylated samples, with a decrease of 34.4% (*P*‐value < 0.01) (Fig. 7B). Therefore, we concluded that acetylation was responsible for the decrease in activity observed in OPRTase.

### Acetylation in K26 and K103 decreases OPRTase enzymatic activity

**Fig. 8:**
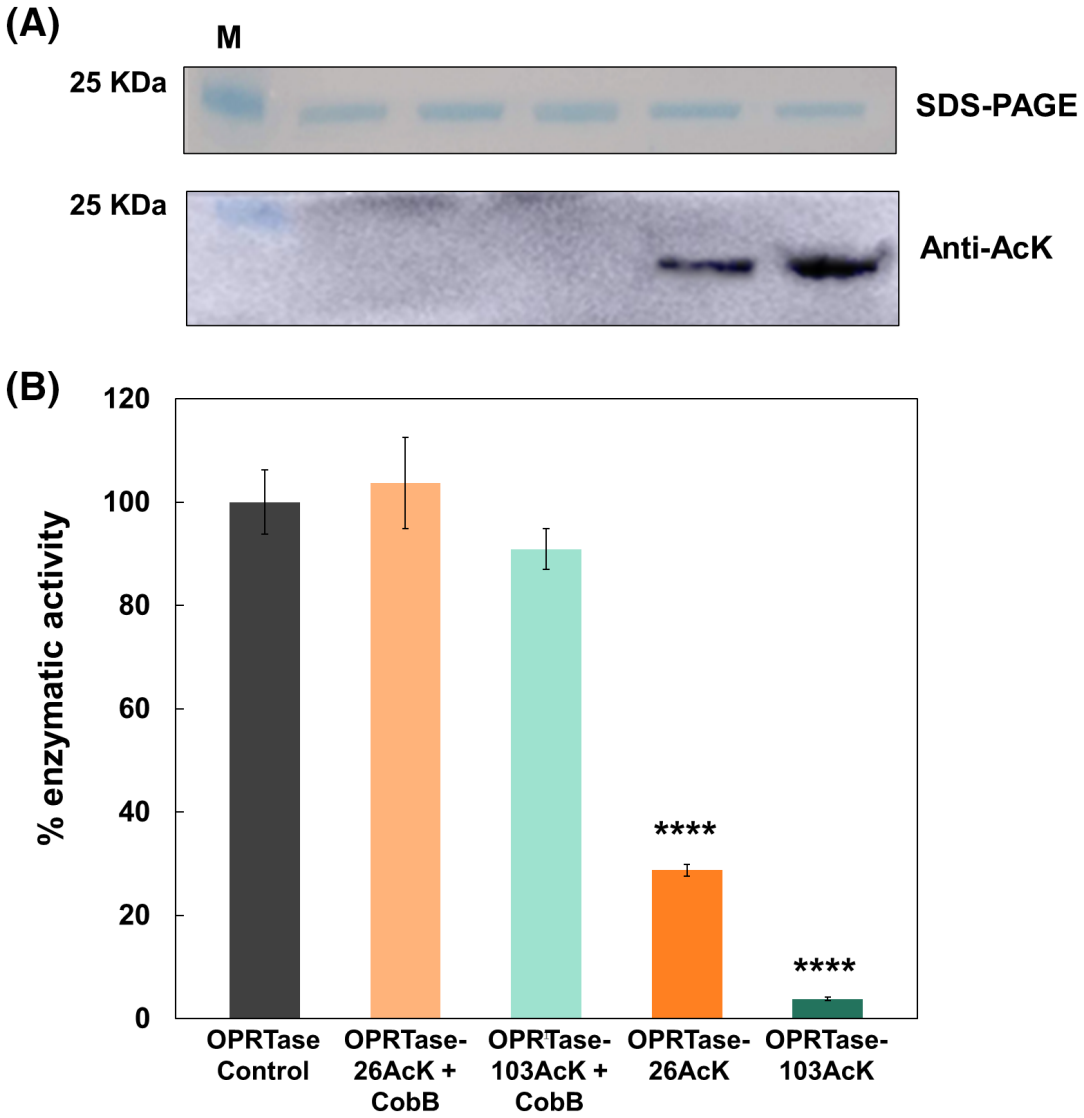
Western blot and enzymatic activity of OPRTase (control), OPRTase‐26AcK subjected to a deacetylation process (OPRTase‐26AcK + CobB), OPRTase‐103AcK subjected to a deacetylation process (OPRTase‐103AcK + CobB), OPRTase‐26AcK and OPRTase‐103AcK. (A) SDS/PAGE and western blot anti‐AcK analysis of OPRTase control (lane 1), OPRTase‐26AcK + CobB (lane 2), OPRTase‐103AcK + CobB (lane 3), OPRTase‐26AcK (lane 4) and OPRTase‐103AcK (lane 5) samples. The molecular mass marker (M) is included in the left side. (B) Relative enzymatic activities of OPRTase and its variants. OPRTase control, OPRTase‐26AcK + CobB, OPRTase‐103AcK + CobB, OPRTase‐26AcK and OPRTase‐103AcK. The activity of OPRTase control sample was set to 100%. A one‐way ANOVA test was carried out to identify significant differences between relative enzymatic activities of the samples with respect to the control sample [*P*‐value < 0.0001 (****)]. Error bars are standard errors calculated from three repeats. For these assays proteins were purified from cultures of *Escherichia coli* BL21 (DE3) transformed with correspondent vector and grown in LB medium.

Proteomic studies of *E. coli* have detected four lysines subject to acetylation in OPRTase, K19, K26, K103 and K194 [36, 37, 38, 39, 40, 41]. Two of these residues, K26 and K103, have an essential role in OPRTase catalysis in *E. coli* and other organisms in which they are conserved [9, 53, 54]. Hence, we chose these two lysine residues to know the effect of acetylation. To this end, we employed the genetic code expansion approach to site‐specifically incorporate *N*ε‐acetyl‐l‐lysine in these positions [55]. Site‐specifically acetylated proteins were purified in *E. coli* BL21 in LB medium as nonacetylated control OPRTase (Fig. 7). Deacetylation assays were carried out on OPRTase‐26AcK and OPRTase‐103AcK in presence of CobB deacetylase. To test the acetylation level of the samples, we performed a western blot assay (Fig. 8A), which showed no detectable acetylation in control and deacetylated samples, while OPRTase‐26AcK and OPRTase‐103AcK generated a clear signal against anti‐AcK antibody. Thus, the absence of detectable acetylation in the deacetylated samples showed that both acetylated lysines could be targets of CobB deacetylase *in vitro*. In addition, we studied the thermostability of control OPRTase, acetylated OPRTases and deacetylated OPRTases by incubating the proteins at different temperatures for a fixed period of time and measuring their activity. No differences were found in the residual activity derived from thermostability between control, deacetylated and specifically acetylated OPRTases (data not shown).

Forward reaction to produce OMP was measured in control OPRTase, OPRTase‐26AcK and OPRTase‐103AcK (Fig. 8B). The effect of acetylation in OPRTase‐26AcK and OPRTase‐103AcK led to a strong decrease in enzymatic activity with respect to the control sample (*P*‐value < 0.0001) (Fig. 8B). We observed a 71.2% loss of activity for OPRTase‐26AcK, while acetylation at K103 almost eliminated enzymatic activity (loss of 96.2%). After subjecting the samples to the deacetylation reaction with CobB, we observed a recovery of the activity in both cases, without differences with the activity of the OPRTase control. The results demonstrated the importance of these residues for OPRTase catalytic activity, which is recognized by CobB *in vitro* as substrates.

In order to understand the role of acetylation in K26 and K103 residues, kinetic characterization of OPRTase control and acetylated and deacetylated mutants was carried out for the forward (Table 1A) and reverse reactions (Table 1B). The kinetic parameters of the control OPRTase were in accordance with parameters reported in other studies (Table 1) [56]. Acetylation of lysine 26 involved a 19 and 6.3‐fold decrease in *k*_cat_ in both, forward and reverse reactions (Table 1A,B), with respect to the OPRTase control. By contrast, the *K*_M_ value for PRPP was not affected by K26 acetylation (Table 1A), while *K*_M_ value for OMP increased 4‐fold and *K*_M_ values for orotate and PPi increased 2 and 3‐fold relative to control values (Table 1B). Furthermore, deacetylation of OPRTase‐26AcK entailed a recovery of the OPRTase control kinetic parameters.

**Table 1:** Kinetic parameters of *Escherichia coli* OPRTase control, OPRTases acetylated (OPRTase‐26AcK and OPRTase‐103AcK) and OPRTases subjected to a deacetylation process (OPRTase‐26AcK + CobB and OPRTase‐103AcK + CobB) at 30 °C for the (A) forward reaction and (B) reverse reaction.

Enzyme	*k*_cat_ (s^−1^)	*K*_M_ (μm)	*k*_cat_/*K*_M_ (mm ^−1^·s^−1^)	*K*_M_ (μm)	*k*_cat_/*K*_M_ (mm ^−1^·s^−1^)
(A)		Orotate		PRPP	
OPRTase control	32 ± 2	43 ± 5	739	111 ± 12	286
OPRTase‐26AcK	1.7 ± 0.2	96 ± 10	18	119 ± 9	14
OPRTase‐103AcK	0.11 ± 0.01	44 ± 6	2.6	241 ± 24	0.5
OPRTase‐26AcK + CobB	28 ± 2.3	63 ± 7	450	123 ± 10	228
OPRTase‐103AcK + CobB	25 ± 1.7	46 ± 5	550	140 ± 13	160

(B)		OMP		PPi	
OPRTase control	19 ± 0.7	15 ± 1	1257	36 ± 2	525
OPRTase‐26AcK	3.2 ± 0.2	64 ± 7	50	104 ± 8	31
OPRTase‐103AcK	0.14 ± 0.0	21 ± 2	6.8	115 ± 9	1.2
OPRTase‐26AcK + CobB	17 ± 0.2	14 ± 1	1215	55 ± 6	310
OPRTase‐103AcK + CobB	18 ± 0.2	18 ± 2	985	40 ± 4	457

Regarding acetylation in lysine 103, it resulted in the most dramatic reductions in the turnover number and *k*_cat_/*K*_M_ values. *k*_cat_ values for forward and reverse reactions were 0.11 and 0.14 s^−1^, respectively, which represented a decrease of 300 and 150‐fold. Regarding *K*_M_ values, less pronounced differences were observed than for *k*_cat_ and *k*_cat_/*K*_M_ values. Increases of 2 and 3‐fold were detected for PRPP and PPi *K*_M_ values in OPRTase‐103AcK with respect to OPRTase control, while no differences were detected for orotate and OMP *K*_M_ values. As for K26 mutant, the deacetylation of lysine 103 led to a restoration of the kinetic parameters (Table 1).

### OPRTase *in vivo* acetylation depends on acetyl‐P availability

**Fig. 9:**
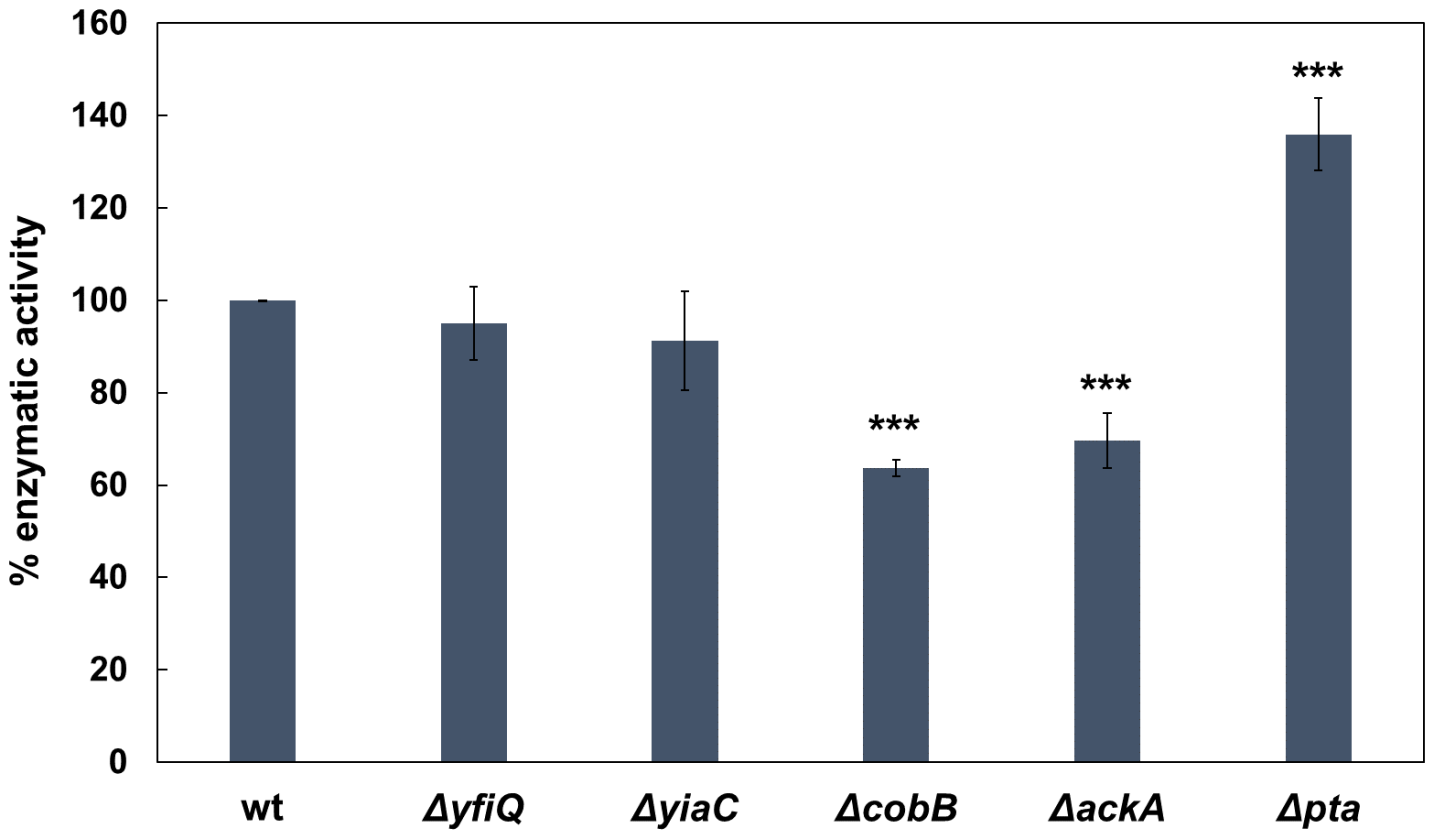
Relative enzymatic activities of OPRTase purified from *Escherichia coli* BL21 wt, Δ*yfiQ*, Δ*yiaC*, Δ*cobB*, Δ*ackA* and Δ*pta*. The activity of OPRTase from wt was set to 100%. A one‐way ANOVA test was carried out to identify significant differences between relative enzymatic activities of the samples with respect to the OPRTase from the wt sample [*P*‐value < 0.001 (***)]. Error bars are standard errors calculated from three repeats. For these assays, proteins were purified from cultures of *E. coli* BL21 (DE3) wt, ΔyfiQ, ΔyiaC, ΔcobB, ΔackA and Δpta strains transformed with pRSET‐pyrE vector and grown in TB7 medium supplemented with 20 mm glucose.

To understand the *in vivo* mechanism leading to the acetylation of OPRTase and the consequent loss of activity, we carried out the purification of the protein from different mutant strains. Some studies have observed that a global increase in lysine acetylation in *E. coli* by an elevated acetyl‐P level is associated with the glucose employ [36, 38]. Therefore, OPRTase was purified from *E. coli* BL21 wt, Δ*yfiQ*, Δ*yiaC*, Δ*cobB*, Δ*ackA* (acetate kinase) and Δ*pta* (phosphate acetyltransferase) growing in TB7 medium supplemented with glucose 20 mm, and enzymatic activity of the forward reaction was measured (Fig. 9). The activity of OPRTase purified from Δ*cobB*, Δ*ackA* and Δ*pta* strains showed significant differences with respect to OPRTase from wt (*P*‐value < 0.001). OPRTase purified from a high acetylation level background (∆*ackA*) showed a reduction in the activity of 30% with respect to OPRTase from wt, and OPRTase from a strain with a low acetylation level (Δ*pta*) showed an increase in activity of 35% relative to OPRTase from wt. By contrast, no significant differences were observed in enzymatic activity of OPRTase purified from Δ*yfiQ* and Δ*yiaC* with respect to OPRTase from wt. The deletion of the only known deacetylase of *E. coli* involved a reduction of 36% in activity relative to OPRTase from wt. To verify that this decrease in activity was due to the acetylation of some of its lysines, purified OPRTase from *cobB* mutant was analysed by LC–MS/MS detecting only K26 acetylated.

### *In vivo* K26 and K103 acetylation hinders a normal physiological behaviour of *E. coli* cells

**Fig. 10:**
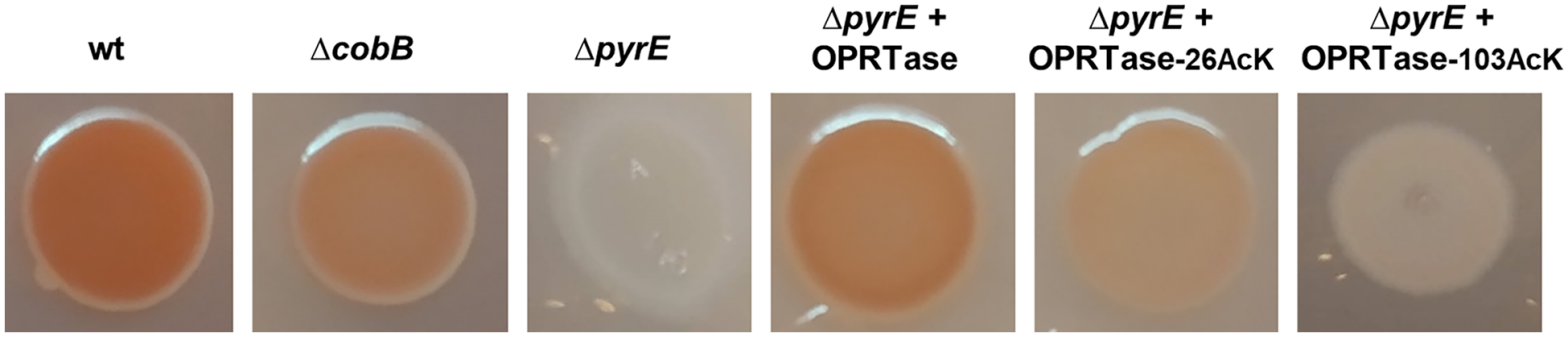
Congo red binding assay of *Escherichia coli* K12 wt, Δ*cobB*, Δ*pyrE*, Δ*pyrE* + OPRTase, Δ*pyrE* + OPRTase‐26AcK and Δ*pyrE* + OPRTase‐103AcK. Strains were spotted in CR medium and grown at 30 °C for 20 h. Plate was incubated at 4 °C for 48 h.

**Fig. 11:**
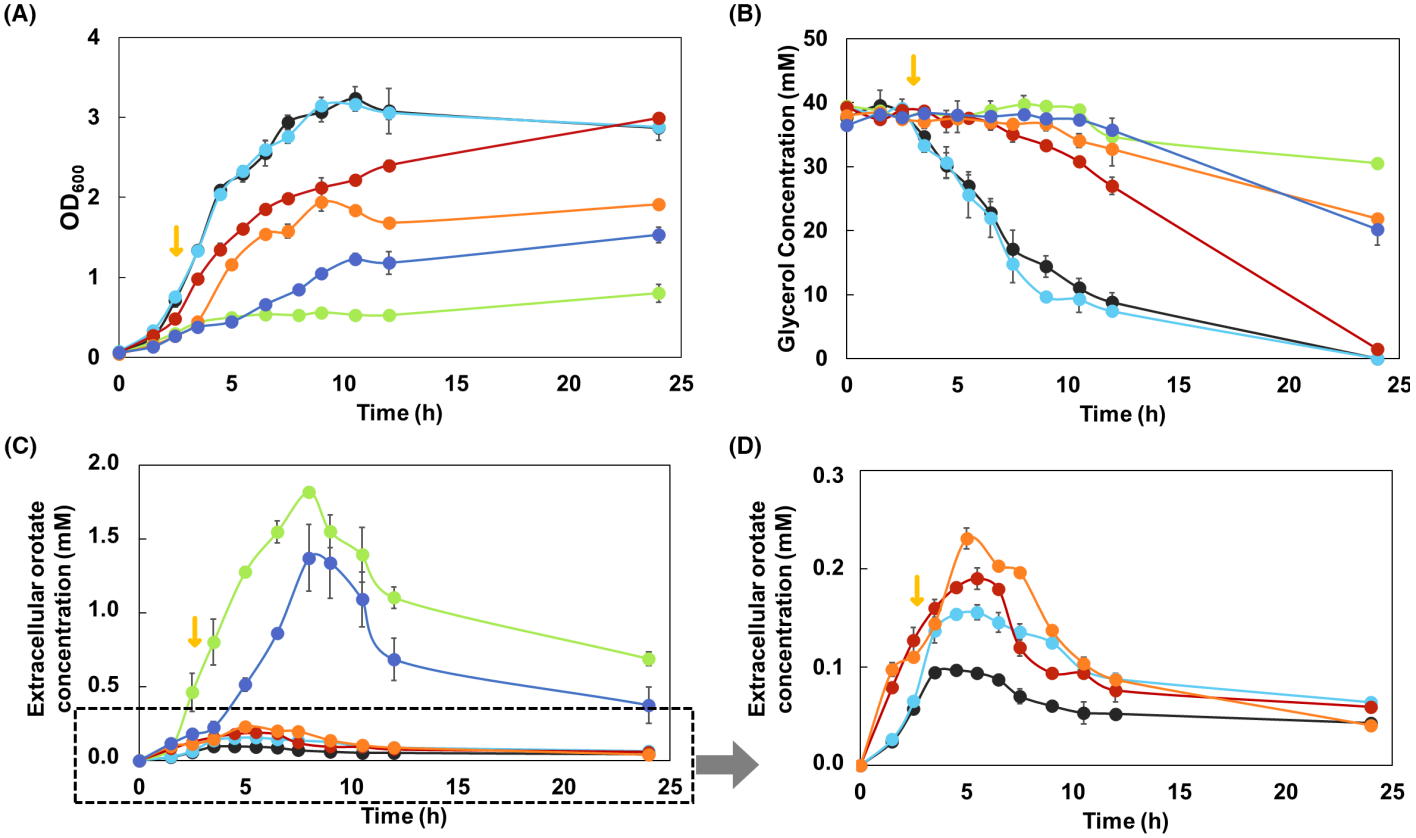
Cell growth at OD_600_ (A), extracellular glycerol (B) and orotate (C, D; 11D presents 11C data that are marked in the box with an altered Y‐axis to better look at specific strains) concentration. *Escherichia coli* K12 wt (black circle), Δ*cobB* (light blue circle), Δ*pyrE* (green circle), Δ*pyrE* + OPRTase (red circle), Δ*pyrE* + OPRTase‐26AcK (orange circle) and Δ*pyrE* + OPRTase‐103AcK (blue circle) growing in TB7 supplemented with glycerol. The yellow arrow indicates the induction time. Error bars are standard errors calculated from three repeats.

To know the physiological consequences of permanent acetylation at lysines 26 and 103 of OPRTase *in vivo*, we carried out a complementation assay of a deficient strain in the *pyrE* gene with P_BAD_ plasmids containing OPRTase control, OPRTase‐26AcK and OPRTase‐103AcK. For this purpose, *E. coli* K12 wt, Δ*cobB*, Δ*pyrE*, Δ*pyrE* + OPRTase, Δ*pyrE* + OPRTase‐26AcK and Δ*pyrE* + OPRTase‐103AcK were grown in TB7 medium supplemented with glycerol 40 mm and overexpression was induced with arabinose. CR medium phenotype (Fig. 10), physiological characterization (Table 2) and extracellular orotate (Fig. 11) were studied.

**Table 2:** Stoichiometric parameters. Specific growth rates (μ_max_), biomass yields (*Y*_X/S_) and specific carbon consumption rates (*q*_s_) of *Escherichia coli* K12 wt, Δ*cobB*, Δ*pyrE*, Δ*pyrE* + OPRTase, Δ*pyrE* + OPRTase‐26AcK and Δ*pyrE* + OPRTase‐103AcK growing in TB7 with glycerol.

	μ_max_ (h^−1^)	*Y*_X/S_ (g·mmolC^−1^)	*q*_s_ [mmolC·(gh)^−1^]
wt	0.988 ± 0.050	0.0107	−64.710
Δ*cobB*	0.959 ± 0.005	0.0095	−63.484
Δ*pyrE*	0.418 ± 0.008	0.0031	−22.516
Δ*pyrE* + OPRTase	0.850 ± 0.028	0.0074	−51.940
Δ*pyrE* + OPRTase‐26AcK	0.652 ± 0.026	0.0039	−31.119
Δ*pyrE* + OPRTase‐103AcK	0.543 ± 0.018	0.0034	−32.388

CR binding assay showed a darker CR medium phenotype for wt strain than for Δ*cobB* strain (Fig. 10), and a white phenotype was observed for Δ*pyrE* strain, indicating starvation in the pyrimidine biosynthesis pathway. The complementation of Δ*pyrE* strain with OPRTase entailed an increase in red phenotype, recovering curli formation at the level of wt strain. However, complementation of Δ*pyrE* strain with OPRTase‐26AcK and OPRTase‐103AcK did not show the dark phenotype, although there was an increase in the formation of curli with respect to the Δ*pyrE* strain. This slight rise in red phenotype was higher in the strain overexpressing OPRTase‐26AcK than with OPRTase‐103AcK, consistent with the higher activity observed for OPRTase‐26AcK than for OPRTase‐103AcK *in vitro*.

To analyse physiological parameters, specific growth rates (μ_max_), biomass yields (*Y*_X/S_) and specific carbon consumption rates (*q*_s_) for all strains were determined (Table 2). These data corroborated the physiological effects obtained in each supplementation test, with similar values for the wt and Δ*cobB* strains, and a reduction of these values for the Δ*pyrE* strain. Supplementation of the Δ*pyrE* strain with the acetylated enzymes showed very similar values to the uncomplemented strain. Moreover, to study the implication of acetylation of these residues in cell growth, extracellular orotate and glycerol concentrations were measured (Fig. 11) identifying several differences between the strains. In the wt strain, glycerol was totally consumed and orotate excretion was the lowest of all tested strains, with a maximum of 0.09 mm (Fig. 11B–D). Regarding the Δ*cobB* strain, orotate excretion was 1.7‐fold higher relative to the wt strain (Fig. 11C,D). Deletion of the *pyrE* gene led to a deep change in cell growth profile and extracellular orotate concentration. This strain only grew to an OD_600_ of 0.8, which is 4‐fold lower than the wt strain, and barely consumed any glycerol, only 22.8% of the total (Fig. 11A,B). The most drastic difference in this strain was in the orotate excretion, which was 19‐fold higher than the excretion observed in wt, with a maximum of 1.82 mm (Fig. 11C). Complementation of Δ*pyrE* strain with OPRTase protein implied the recovery of a profile like that of the wt strain. After induction of OPRTase expression at 3 h of culture, there was an increase in cell growth and the consumption of the carbon source, reaching the level of the wt strain at 24 h of culture (Fig. 11A,B). The greatest change in Δ*pyrE* strain after OPRTase complementation was observed in the extracellular orotate concentration, as the maximum concentration was 0.19 mm, which is 10‐fold lower than without OPRTase supplementation (Fig. 11C,D). On the contrary, complementation of Δ*pyrE* strain with acetylated OPRTase mutant at lysine 103 showed almost the same behaviour as Δ*pyrE* strain alone, involving low cell growth and incomplete consumption of the carbon source (Fig. 11A,B). In addition, supplementation of the Δ*pyrE* strain with the OPRTase‐103AcK protein produced an orotate excretion very similar to that of the uncomplemented strain, with a maximum of 1.37 mm, 15‐fold higher than that of the wt strain (Fig. 11C). Likewise, complementation of the *pyrE* deficient strain with OPRTase‐26AcK showed similar behaviour to complementation with the OPRTase‐103AcK protein. Thus, glycerol was not completely consumed (Fig. 11B), while cell growth was higher than in the Δ*pyrE* strain complemented with OPRTase‐103AcK and the excretion of orotate maximum concentration was 0.24 mm, only 2.7 times higher than for the wt strain (Fig. 11A,C,D). All these results, together with the CR assay and stoichiometric parameters (Table 2), highlighted the low activity of the acetylated OPRTase protein at lysines 26 or 103 *in vivo*, and the relevance of these acetylations for physiological parameters and normal cell development.

## Discussion

The hypothesis of regulation by acetylation of this pathway was based on a previous study in which we observed a 35‐fold increase in the orotate extracellular concentration in the Δ*cobB* with respect to the wt strain in *E. coli* BL21 [50]. In that study, we assumed that the increase in the acetylation level of isocitrate lyase in Δ*cobB* strain could imply a decrease in the flux through the glyoxylate shunt, and therefore increased the flux through the TCA cycle involving a rise in the α‐ketoglutarate (α‐KG) concentration [57]. However, this fact need not be the only cause of the high orotate extracellular concentration in Δ*cobB* mutant. In the present study, the darker CR medium phenotype showed by the wt strain with respect to Δ*cobB* strain (Fig. 2) indicated a lower pyrimidine biosynthesis in the *cobB* deficient strain. Therefore, this fact together with the accumulation of orotate in Δ*cobB* strain could indicate a blockage of the route by acetylation. In addition, some proteins of the pathway have been identified as CobB interactors [58] and appear acetylated in diverse *E. coli* acetylome studies [36, 37, 38, 39, 40, 41].

To determine whether there was a blockage of the pyrimidine biosynthesis pathway by lysine acetylation, the concentration of intracellular and extracellular metabolites was studied in wt and Δ*cobB* strains, and the results were derived from it reinforced our hypothesis (Figs 3, 4, 5). The final product concentration of the route and precursor of all pyrimidines, UMP, was significantly higher in the wt than in Δ*cobB* strain (Fig. 3), which was consistent with results from the CR medium (Fig. 2). Despite the lower final concentration of UMP in the Δ*cobB* strain, we observed two metabolites in which the concentration in this mutant was significantly greater than in wt strain, CP and orotate (Fig. 3). These data would support an OPRTase regulation by acetylation. Subsequently, western blot and enzymatic activity assays of OPRTase purified, deacetylated with CobB and chemically acetylated with 10 mm acetyl‐P were carried out (Fig. 7). Deacetylated OPRTase, checked by western blot (Fig. 7A), involved a slight increase of enzymatic activity (Fig. 7B), while acetylation led to a significant decrease in the activity (Fig. 7B). Moreover, through site‐specifically incorporation of *N*ε‐acetyl‐l‐lysine in K26 or K103 positions of the OPRTase protein we provided biochemical evidence for the effect of acetylation/deacetylation on OPRTase enzyme activity. Enzymatic activity was strongly affected by acetylation in K26 and K103 with a loss of 71.2% and 96.2%, respectively, which implies almost the disappearance of the enzymatic activity in OPRTase‐103AcK (Fig. 8). Deacetylation of OPRTase‐26AcK and OPRTase‐103AcK enzymes *in vitro* reverted the changes produced, recovering enzyme activity and kinetic parameters at values like those for OPRTase control (Fig. 8 and Table 1).

**Fig. 12:**
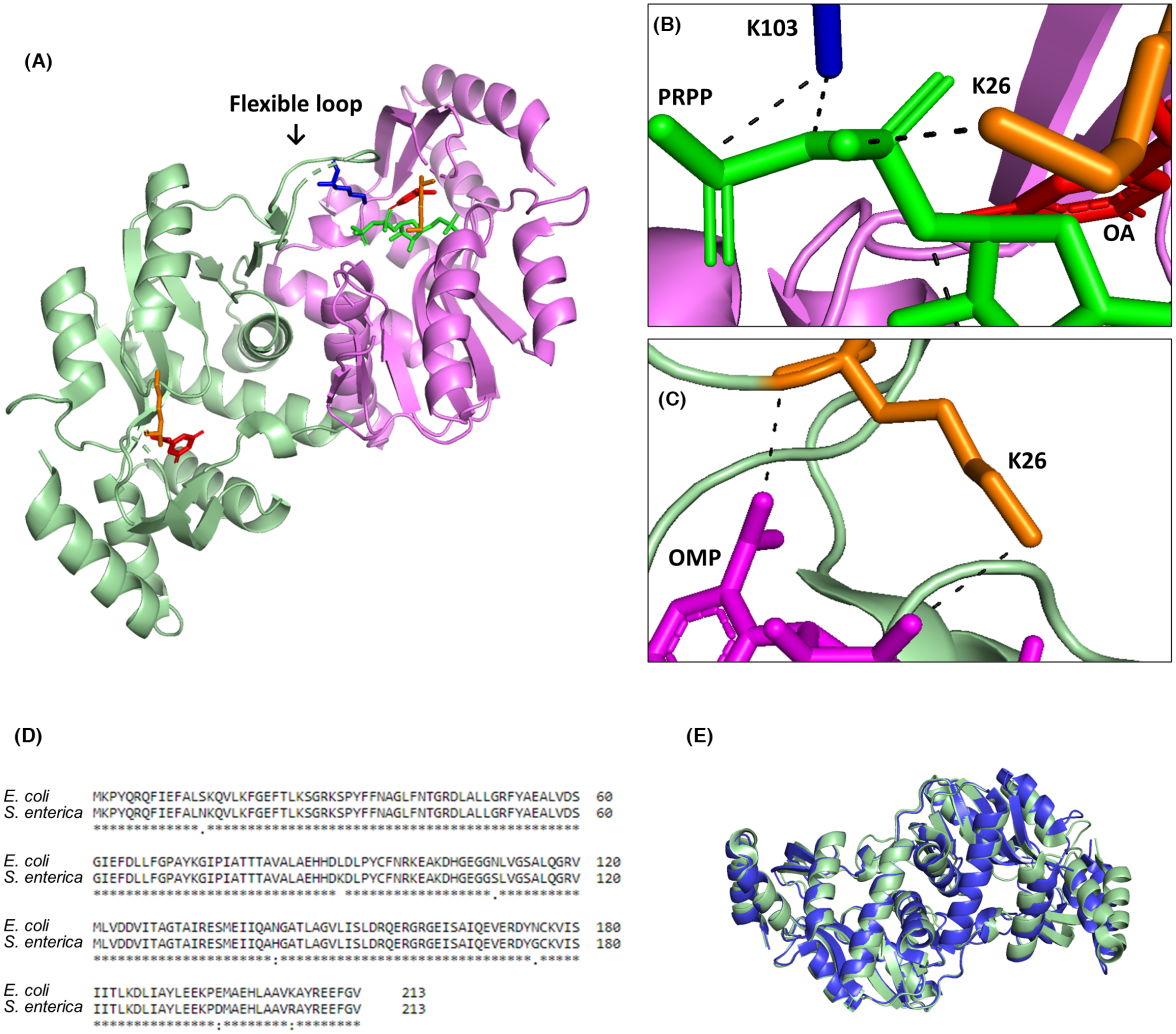
Crystallographic structure of *Salmonella enterica* OPRTase. (A–C) Two subunits were coloured green and purple separately. K103 is marked in blue and K26 is marked in orange. (A, B) OPRTase with PRPP (green) and orotate (red) (PDB ID: 1LH0). (C) OPRTase with OMP (purple) (PDB ID: 1STO). Hydrogen bonds found between lysines ${\mathrm{NH}}_{3}^{+}$ groups, K26 or K103, and the substrates PRPP and OMP are shown in dashed lines. (D) Protein sequence alignment of OPRTases from *Escherichia coli* (Uniprot ID: P0A7E3) and *S. enterica* (Uniprot ID: P08870). (E) Alignment of crystallographic structures of *E. coli* (green) (PDB ID: 6TAK) and *S. enterica* (blue) (PDB ID: 1LH0) OPRTases. The reported structures have been generated using pymol Software (DeLano Scientific LLC, San Francisco, CA, USA), and the alignment has been made employing clustal omega from the EMBL‐EBI (Cambridge, UK).

Several studies have demonstrated the importance of these lysine residues in the OPRTases of *E. coli* and other microorganisms [9, 24, 29, 54, 59], but the acetylation effect on K26 and K103 had not been studied, to our knowledge, yet. *E. coli* and *Salmonella enterica* OPRTases share more than 97% of identity in their sequences and the same overall fold [9, 59] (Fig. 12D,E), so we have used *S. enterica* structures to show the effects of acetylation (Fig. 12A–C). The vast majority of the structures of the reported OPRTases are composed of two subunits with a flexible loop that sits on the catalytic site of the adjacent subunit to interact with the PRPP substrate [54, 59, 60, 61]. Attending to the position and interactions of the lysine residues studied, K26 has been reported to be involved in OMP, orotate and, to a lesser extent, PRPP binding [53]. The nitrogen backbone of K26 forms a hydrogen bond with the carboxylate group of OMP and orotate, and the ε‐amino group of K26 forms hydrogen bonds with a hydroxyl group of OMP and with the 5′‐phosphate moiety of PRPP [9, 29, 54, 59, 62] (Fig. 12A–C). Therefore, acetylation of the ε‐amino group of K26 would be responsible for some distortion of the architecture and electrostatic charge in the enzyme active centre, resulting in an increase in *K*_M_ for orotate, PPi and OMP, and a decrease in *k*_cat_ from 6 and 19‐fold for reverse and forward reaction, respectively (Table 1). These results were similar to those obtained in other studies for the mutation of the lysine at this position by glutamine or alanine [63]. Regarding OPRTase‐103AcK, the covalent bonding of the acetyl radical to the ε‐amine of the lysine residue involved the largest catalytic changes, since the enzymatic activity was strongly affected with a reduction of *k*_cat_ of 300 and 150 times for back and forth reaction and increases were observed in *K*_M_ for the substrates PRPP and PPi (Table 1). Furthermore, these results confirm the essential role of K103 for catalysis, previously observed when lysine was substituted by glutamine or alanine [63]. K103 belongs to the highly conserved catalytic loop of OPRTase (residues 99–109), which remains in an open conformation without the substrate and moves to a close conformation to interact with bound substrates and sequester them from bulk solvent in the adjacent subunit [9, 59, 60]. Hence, K103 moves to the adjacent subunit catalytic site and interacts with the pyrophosphate moiety of PRPP, makes an intra‐loop hydrogen bond with the carboxylate of E107 and helps to release PPi after catalytic turnover through the ε‐amino group, and in addition, K103 binds to G109 carbonyl oxygen through backbone nitrogen [9, 29, 54, 59, 61] (Fig. 12A–C). Consequently, the increase in the size of side chain and the neutralization of positive charge caused by K103 acetylation would involve an inefficient charge distribution and loop folding and, therefore, an impediment to the development of catalytic activity as we observed in this work (Fig. 8 and Table 1).

In addition, *in vivo* lysine acetylation of *E. coli* OPRTase was demonstrated since protein purified from mutants related to this modification showed different enzymatic activity. Specifically, OPRTase activity decreased when was expressed in ∆*ackA* or ∆*cobB* strains and increased in ∆*pta* strain (Fig. 9). The results suggest that OPRTase acetylation could derive from the build‐up of metabolic intermediates, mainly acetyl‐P, paralleled glucose consumption, which favours nonenzymatic acetylation [36, 38]. Results were in accordance with the proteome studies in which OPRTase has been found acetylated at lysine residues 26 and 103 under conditions that favour chemical acetylation, such as with a high concentration of glucose as a carbon source and in the stationary growth phase [36, 38, 39, 40]. Moreover, no significant differences were observed in enzymatic activity of OPRTase purified from wt, Δ*yfiQ* and Δ*yiaC*. In addition, lysines 26 and 103 did not appear as substrates for YfiQ, YiaC and other new KATs (RimI, YjaB and PhnO) in a recent study in which these KATs were overexpressed [37]. However, it cannot be ruled out that another acetyltransferase could be involved in regulating OPRTase acetylation.

Physiological effects of the inactivation of OPRTase due to the acetylation of lysines were shown in Figs 10 and 11 and Table 2. Complementation of Δ*pyrE* mutant with OPRTase returned a similar profile to wt strain (Figs 10 and 11 and Table 2). As regards permanent acetylation in K103 of OPRTase, it resulted in an analogous behaviour to that noticed for Δ*pyrE* strain (Figs 10 and 11 and Table 2). These results implied that OPRTase‐103AcK was not active enough *in vivo* to complement the deletion of the *pyrE* gene. For its part, acetylation of K26 in OPRTase prevented it from the restoration of wt phenotype. Nevertheless, the enzyme was less affected by this acetylation than by acetylation at position 103, since pyrimidine biosynthesis and cell growth were higher, and orotate excretion was significantly lower (Figs 10 and 11 and Table 2). Finally, the implication of permanent acetylation, of both K26 and K103, in physiological behaviour and orotate excretion profile was greater than that derived from *cobB* deletion. This could be because in the ∆*cobB* strain the acetylation of these lysines is not as complete and homogeneous as that of the mutant proteins OPRTase‐26AcK and OPRTase‐103AcK.

**Fig. 13:**
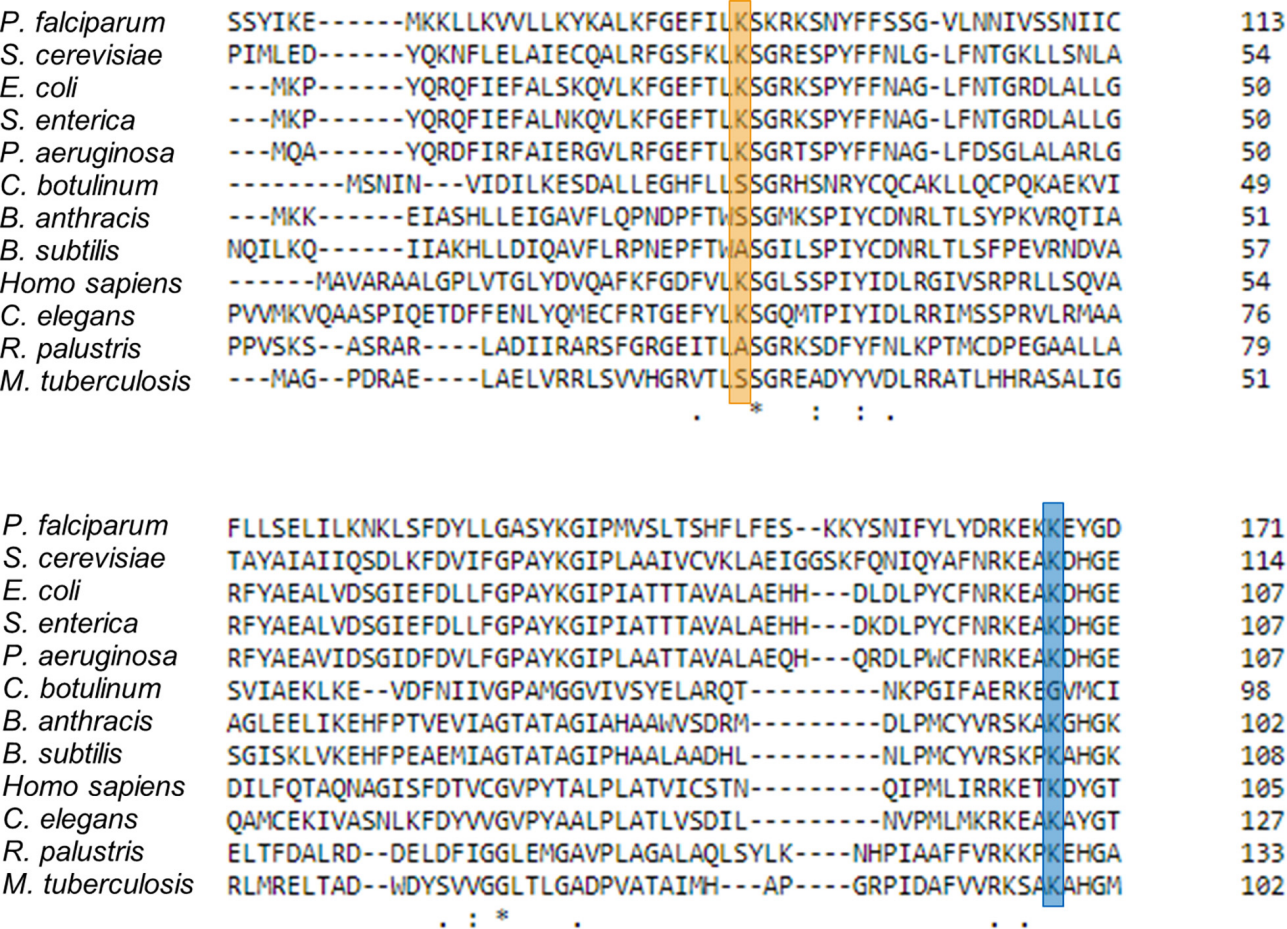
Protein sequence alignment of OPRTases from different eukaryotes and prokaryotes organisms. *Escherichia coli* (Uniprot ID: P0A7E3), *Plasmodium falciparum* (Uniprot ID: Q8I3Y0), *Saccharomyces cerevisiae* (Uniprot ID: P13298), *Salmonella enterica* (Uniprot ID: P08870), *Pseudomonas aeruginosa* (Uniprot ID: P50587), *Clostridium botulinum* (Uniprot ID: A5I6W5), *Bacillus anthracis* (Uniprot ID: A0A640L9R9), *Bacillus subtilis* (Uniprot ID: P25972), *Homo sapiens* (Uniprot ID: P11172), *Caenorhabditis elegans* (Uniprot ID: O61790), *Rhodopseudomonas palustris* (Uniprot ID: Q07H43) and *Mycobacterium tuberculosis* (Uniprot ID: P9WHK9). Orange box indicates residue K26, and blue box indicates residue K103 of *E. coli* OPRTase. The alignment has been made employing clustal omega from the EMBL‐EBI.

It is remarkable that K26 and K103 residues are widely conserved among OPRTases in different organisms. Protein alignment of OPRTase sequences of diverse important prokaryotes and eukaryotes organisms employed as a model or responsible for the development of various diseases (*E. coli*, *Plasmodium falciparum*, *Saccharomyces cerevisiae*, *S. enterica*, *Pseudomonas aeruginosa*, *Clostridium botulinum*, *Bacillus anthracis*, *Bacillus subtilis*, *Homo sapiens*, *Caenorhabditis elegans*, *Rhodopseudomonas palustris* and *Mycobacterium tuberculosis*) showed that K103 is conserved in all organisms except for *C. botulinum*, while K26 is less conserved because it is not found in some Gram‐positive bacteria and in *R. palustris* (Fig. 13).

In addition to the importance of the conservation of these lysines, the relevance of this work lies in the fact that the pathway to which OPRTase belongs, and the enzyme itself, is key to the development of certain human pathologies and, therefore, interesting targets for their treatment. Thus, this enzyme is of interest for the treatment of tuberculosis caused by *M. tuberculosis* and malaria caused by *P. falciparum* [24, 25]. Specifically, in studies on the acetylome of this parasite, the conserved lysine homologous to *E. coli* lysine 26 has been detected acetylated [64]. On the other hand, the pyrimidine biosynthesis pathway is also a requirement for the proliferation of pathogens such as *E. coli*, *S. enterica* and *B. anthracis* in blood [65], for the colonization of the urinary tract by *E. coli* [66] and for the colonization of the intestine by *E. coli* and *S. enterica* [67, 68]. In addition, this pathway is important for virulence, cytotoxicity and antibiotic resistance of *P. aeruginosa*, and deletion of *pyrE* has been reported to cause a decrease in virulence factors of this microorganism [69]. However, pyrimidine biosynthesis is not only important for pathogenic organisms, but it has also been observed that it is relevant to consider the microbiota for the metabolism of different drugs [70]. For example, OPRTase in the microbiota helps the metabolism of a chemotherapeutic drug [71]. At the same time, it has been recently reported that the acetylation pattern of the microbiota may change as a consequence of different diseases [72].

The results of this work reveal a crucial posttranslational regulation of the pyrimidine biosynthesis pathway by lysine acetylation of OPRTase, the fifth enzyme of the pathway. In addition, lysine acetylation in K26 or K103 residues of OPRTase, presumably by a chemical mechanism, can be reverted by *E. coli* sirtuin CobB, which gives a greater dimension to the regulatory nature of this modification. Although this enzyme has been modified by other PTMs such as succinylation [73] and propionylation [74], this is the first time, to our knowledge, that regulation by lysine acetylation of an OPRTase is reported. Our work demonstrates the regulation of this enzyme by acetylation in *E. coli* may have important consequences for human health through the development of new drugs, and it also represents an important starting point for the investigation of homologous enzymes.

## Materials and methods

### Molecular biology: plasmid construction and incorporation of *N*ε‐acetyl‐lysine into OPRTase at K26 and K103 positions

All strains, plasmids and primers employed in this study are listed in Table S1. All molecular biology enzymes employed were purchased from Thermo Fisher Scientific (Waltham, MA, USA). *E. coli* K12 BW25113 Δ*pyrE* and *E. coli* BL21 (D3) Δ*yiaC* knockout strains were constructed employing the phage lambda Red recombinase method [75]. To overexpress the OPRTase protein, the *pyrE* gene was inserted into pRSET‐A or pET28a‐*mbp* plasmids, to construct pRSET‐*pyrE* and pET28a*‐mbp‐pyrE*.

To site‐specifically incorporate *N*ε‐acetyl‐l‐lysine in K26 or K103 positions of the OPRTase protein the expansion code concept was employed. For this purpose, modified vector pRSF‐Duet‐1 [55] (kindly supplied by M. Lammers, University of Cologne) encoding for the synthetically evolved acetyl‐lysyl‐tRNA‐synthetase, AcKRS3 and the amber suppressor tRNA_CUA_, *Mb*tRNA_CUA_ derived from *Methanosarcina barkeri*, was used. *pyrE* gene was modified in K26 or K103 by directed mutagenesis from pET28a‐*mbp*‐*pyrE* vector to incorporate an amber stop codon in these positions. The *mbp‐pyrE* ^26amber^ and *mbp‐pyrE* ^103amber^ genes were PCR amplified from pET28a‐*mbp*‐*pyrE* ^26amber^ and pET28a‐*mbp*‐*pyrE* ^103amber^ and cloned into pRSF‐Duet‐1‐acetyl lysyl‐tRNA‐synthetase AcKRS3/*Mb*tRNA_CUA_, to obtain pRSF‐*mbp*‐*pyrE* ^26AcK^ and pRSF‐*mbp*‐*pyrE* ^103AcK^.

For the complementation of *E. coli* K12 Δ*pyrE* with OPRTase, OPRTase‐26AcK and OPRTase‐103AcK proteins, plasmids with P_BAD_ promoter positively induced by l‐arabinose were constructed (pSF‐pMB1′‐BAD‐*mbp‐pyrE*, pRSF‐BAD‐*mbp*‐*pyrE* ^26AcK^ and pRSF‐BAD‐*mbp*‐*pyrE* ^103AcK^). The *mbp‐pyrE* gen and pSF‐pMB1′‐BAD‐YFP vector [76] were PCR amplified to construct pSF‐pMB1′‐BAD‐*mbp‐pyrE* plasmid by sequence and ligation‐independent cloning (SLIC) [77]. To construct the other two plasmids, the P_BAD_ promoter and *araC* regulatory from pBAD24 plasmid, and pRSF‐*mbp*‐*pyrE* ^26AcK^ and pRSF‐*mbp*‐*pyrE* ^103AcK^ vectors were PCR amplified. The pRSF‐BAD‐*mbp*‐*pyrE* ^26AcK^ and pRSF‐BAD‐*mbp*‐*pyrE* ^103AcK^ vectors were constructed by SLIC.

### Congo red binding assay

*Escherichia coli* K12 wild‐type (wt), *E. coli* Δ*cobB* and *E. coli* Δ*pyrE* strains transformed with pSF‐pMB1′‐BAD‐*mbp‐pyrE*, pRSF‐BAD‐*mbp*‐*pyrE* ^26AcK^ and pRSF‐BAD‐*mbp*‐*pyrE* ^103AcK^ vectors were grown overnight at 37 °C in TB7 (10 g·L^−1^ tryptone buffered at pH 7.0 with 100 mm potassium phosphate) supplemented with glycerol 40 mm. Glycerol was used for the correct protein expression with the P_BAD_ promoter. Protein expression was induced for 8 h by the addition of arabinose 2 mm and for acetylated protein expression, nicotinamide 20 mm and *N*ε‐acetyl‐l‐lysine 10 mm were also added. An aliquot of 5 μL of each strain culture was spotted on YESCA plates (yeast extract 0.5 g·L^−1^, casamino acids 10 g·L^−1^, and agar 20 g·L^−1^) supplemented with Congo red 30 μg·mL^−1^ and Coomassie Blue 10 μg·mL^−1^ (CR medium). Plates were incubated for 20 h at 30 °C and dye‐binding was better detected after incubation at 4 °C for 48 h. When needed, ampicillin 100 μg·mL^−1^ or kanamycin 30 μg·mL^−1^ was added.

### Intracellular and extracellular *de novo* pyrimidine biosynthesis metabolites quantification

*Escherichia coli* K12 wt and *E. coli* K12 Δ*cobB* strains were grown in duplicate in batch mode at 37 °C with shaking (250 r.p.m.) in minimal M9 medium (10 mm (NH_4_)_2_SO_4_, 8.5 mm NaCl, 40 mm Na_2_HPO_4_, 20 mm KH_2_PO_4_, 185 μm FeCl_3_, 175 μm EDTA, 7 μm ZnSO_4_, 7 μm CuSO_4_ 5 H_2_O, 7 μm MnSO_4_, 7 μm CoCl_2_, 1 mm MgSO_4_, 0.1 mm CaCl_2_, and 1 μm thiamine HCl) supplemented with glucose 20 mm. A minimal M9 medium was selected to reduce possible interferences in the subsequent analysis of the samples. In addition, induction of acetylation needs glucose, so we used glucose as a carbon source and minimal medium instead of complex [36, 38]. For the quenching procedure, the method developed by Spura et al. [78] was employed. Samples of 20 mL of culture at different growth times were rapidly transferred to a precooled (−20 °C) 50 mL Falcon tube with 20 mL of quenching solution [ethanol 40% (v/v) and NaCl 0.8% (w/v)] and cooled to approximately −5 °C in a bath with ethylene glycol solution at −20 °C. Subsequently, samples were centrifuged in a Sigma 6‐16K centrifuge (12169‐H rotor, *r* = 10 cm) (Sigma Laborzentrifugen GmbH, Osterode am Harz, Germany) at −11 °C for 5 min at 2500 ***g***. Cell pellets were immediately frozen in liquid nitrogen. Metabolite extraction from cell pellet was carried out by a freeze–thaw method with acetonitrile/methanol/water (2 : 2 : 1) at −20 °C [79]. Pellets were resuspended in 1 mL of extraction buffer and subjected to three freeze–thaw cycles (liquid N_2_‐ethylene glycol bath at −20 °C), and then, samples were centrifuged at −9 °C for 10 min at 25 000 ***g***. Subsequently, the supernatant was evaporated to dryness with a speed‐vac and the residue was stored until the analysis platform was ready, to avoid potential metabolic degradation. Then, residues were resuspended in 0.5 mL of HPLC water (pH 7) per 1 unit of OD_600_ of the original culture, and the autosampler was maintained at 4 °C. On the other hand, to quantify extracellular metabolites, 1 mL of cultures at the same culture phases were taken, and pellets were harvested by centrifugation at 4 °C for 1 min at 16 000 ***g*** and discarded, then supernatants were evaporated in a speed‐vac and resuspended in HPLC water prior to analysis.

The separation and analysis of samples were performed with an HPLC/MS system consisting of an Agilent 1290 Infinity II Series HPLC (Agilent Technologies, Santa Clara, CA, USA) connected to an Agilent 6550 Q‐TOF Mass Spectrometer (Agilent Technologies) using an Agilent Jet Stream Dual electrospray (AJS‐Dual ESI) interface. Samples were analysed with two different methods, one for OMP and the other for the rest of the metabolites (CP, l‐arginine, l‐aspartic acid, l‐glutamine, l‐glutamic acid, ATP, CASP, DHO, orotate and UMP).

For all metabolites analysis, samples were injected onto an Agilent Zorbax Eclipse Plus C18 (2.1 × 100 mm, 1.8 μm) HPLC column thermostatted at 40 °C, at a flow rate of 0.4 mL·min^−1^. Solvents A (MilliQ water with 0.1% formic acid) and B (acetonitrile with 0.1% formic acid) were used for the compound separation. The elution programme consisted of 2% phase B for 1 min and then a gradient from 2% to 100% of phase B in 9 min. 100% solvent B was maintained for 2 min and then another gradient from 100% to 2% of phase B in 1 min. Finally, 2% phase B was maintained for two additional minutes. The mass spectrometer was operated in the positive mode. The nebulizer gas pressure was set to 30 psi, whereas the drying gas flow was set to 16 L·min^−1^ at a temperature of 130 °C, and the sheath gas flow was set to 11 L·min^−1^ at a temperature of 300 °C. The capillary spray, nozzle, fragmentor and octopole 1 RF Vpp voltages were 4000, 500, 350 and 750 V, respectively. MS Data scans were acquired in the 50–500 *m*/*z* range in 2 GHz extended dynamic range mode with 4 spectra·s^−1^ and 250 ms·spectrum^−1^. Three collision energies (0, 10 and 40 V) were measured in each cycle. Reference mass at 121.0509 was used for mass correction during the analysis. To analyse OMP, samples were injected onto an Agilent HILIC Plus (4.6 × 100 mm, 3.5 μm) HPLC column, at a flow rate of 0.5 mL·min^−1^. The column was equilibrated at 40 °C. Solvents A (methanol with 5 mm ammonium acetate) and B (5 mm ammonium acetate) were used for the compound separation. The elution programme consisted of 2% phase B for 2 min and then a gradient from 2% to 100% of phase B in 4 min. 100% solvent B was maintained for 1 min and then another gradient from 100% to 2% of phase B in 0.5 min. Finally, 2% phase B was maintained for 1.5 additional minutes. The mass spectrometer was operated in the negative mode. The nebulizer gas pressure was set to 30 psi, whereas the drying gas flow was set to 16 L·min^−1^ at a temperature of 150 °C, and the sheath gas flow was set to 12 L·min^−1^ at a temperature of 300 °C. The capillary spray, nozzle, fragmentor and octopole 1 RF Vpp voltages were 4000, 1000, 360 and 750 V, respectively. MS Data scans were acquired in the 50–500 *m*/*z* range in 2 GHz extended dynamic range mode with 4 spectra·s^−1^ and 250 ms·spectrum^−1^. Three collision energies (0, 10 and 40 V) were measured in each cycle. Reference mass at 112.985587 was used for mass correction during the analysis. Data analysis was performed with masshunter qualitative analysis navigator software (Rev. B.08.00; Agilent Technologies).

**Fig. 14:**
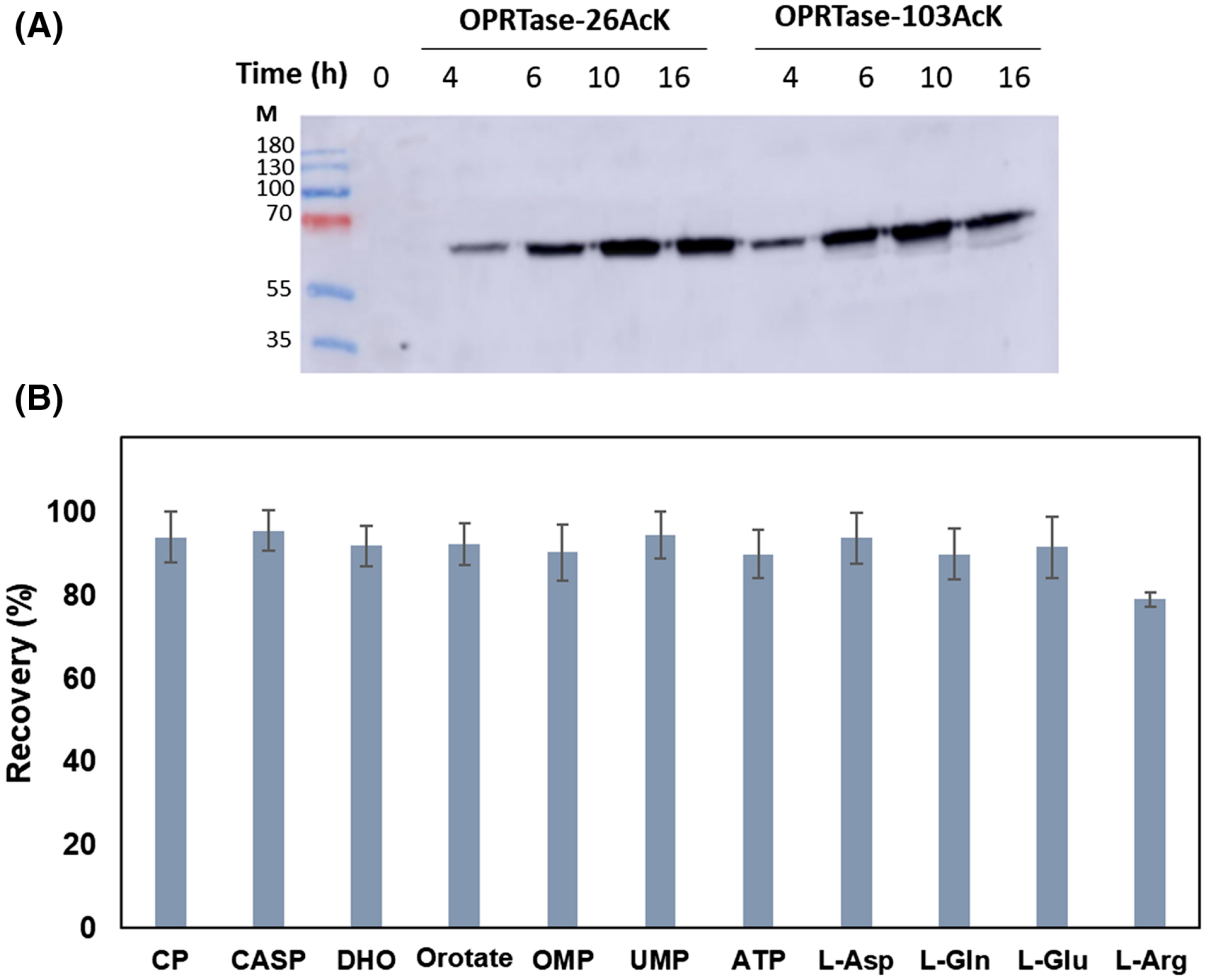
Overexpression western blot and intracellular metabolite recovery. (A) Western blot using anti‐(His)_6_ tag antibody of OPRTase‐26AcK and OPRTase‐103AcK overexpression cultures at different growth time after induction (time 0). For these assays, samples were taken from cultures of *Escherichia coli* BL21 (DE3) transformed with pRSF‐pMB1′‐BAD‐*mbp‐pyrE* ^26AcK^ and pRSF‐pMB1′‐BAD‐*mbp‐pyrE* ^103AcK^ vectors and growing in LB medium supplemented with 10 mm Nε‐acetyl‐l‐lysine and 20 mm nicotinamide 30 min before induction. (B) Intracellular metabolite recovery after the extraction protocol, as described in material and methods section. Error bars are standard errors calculated from three repeats.

The standards of all the compounds studied were subjected to the extraction process to check that compounds were not deteriorated and to quantify the recovery from calibration curves (Fig. 14B). Intracellular metabolite concentrations are given in terms of the volume occupied by cells, estimated from a cell volume of 0.7 μm^3^ and a cell number of 7.5 × 10^8^ cells·mL^−1^ per OD_600_.

### Protein expression and purification

*Escherichia coli* BL21 (DE3) wt strains were transformed with pET28a‐*mbp*‐*pyrE*, pRSET*‐pyrBI*, pET28a‐*mbp*‐*cobB*, pRSF‐*mbp*‐*pyrE* ^26AcK^ or pRSF‐*mbp*‐*pyrE* ^103AcK^ overexpression vectors and grown in Luria‐Bertani broth [(LB) 10 g·L^−1^ tryptone, 5 g·L^−1^ yeast extract, and 5 g·L^−1^ NaCl] at 37 °C with orbital shaking (200 r.p.m.) until OD_600_ reached 0.5–0.6 units, moment in where protein overexpression was induced with 0.2 mm IPTG. LB carbon source‐free was selected because the induction of acetylation was not necessary [36, 80]. For the overexpression of OPRTase‐26AcK and OPRTase‐103AcK proteins, cultures were supplemented with 10 mm *N*ε‐acetyl‐l‐lysine and 20 mm nicotinamide 30 min before induction. After induction, cultures were grown overnight at 20 °C with orbital shaking (200 r.p.m.). Chemically competent *E. coli* BL21 (DE3) wt and Δ*ackA*, Δ*pta*, Δ*yfiQ*, Δ*yiaC* and Δ*cobB* strains were transformed with pRSET‐*pyrE* vector and were grown in TB7 medium supplemented with glucose 20 mm to promote acetylation [36, 38] at 37 °C with orbital shaking (200 r.p.m.). Cultures were induced at 0.6–0.8 OD_600_ units with 0.4 mm IPTG, and then, cultures were grown at 20 °C with orbital shaking (200 r.p.m.) overnight. Cell pellets were harvested by centrifugation at 4 °C for 20 min at 3000 ***g*** and resuspended in binding buffer (50 mm potassium phosphate, 25 mm imidazole, 500 mm NaCl, pH 8). Cells were disrupted on ice by sonication for 2 min (20 s each pulse) employing a Vibra Cell sonicator (Sonicator Sonics & Materials, Newton, UK), and lysates were clarified by centrifugation for 30 min at 4 °C and 16 000 ***g***. Supernatants were loaded onto a Ni (II)‐loaded 5 mL His‐Trap HP column (GE Healthcare, Chicago, IL, USA) previously equilibrated in binding buffer. Proteins were eluted using a linear gradient of imidazole from 0 to 500 mm at a flow rate of 5 mL·min^−1^. The protein buffer was changed to conservation buffer (150 mm Tris–HCl, 50 mm NaCl, pH 7.5) employing a HiPrep™ 26/10 desalting column (GE Healthcare) at a flow rate of 9 mL·min^−1^.

### *In vitro* nonenzymatic acetylation assay and deacetylation assay

*In vitro* nonenzymatic acetylation assay was performed in conservation buffer. Fifty micrograms of enzymes were incubated with acetyl‐P 10 mm at 37 °C for 6 h with orbital shaking (200 r.p.m.) in a total volume of 100 μL. A control reaction under the same conditions but without acetyl‐P was carried out.

The deacetylation reaction was performed in a conservation buffer supplemented with NAD^+^ 1 mm. The assay was carried out by mixing 50 μg of enzymes with 100 μg of CobB in a total volume of 100 μL, the reaction was then incubated for 6 h at 37 °C with orbital shaking (200 r.p.m.). A control reaction without NAD^+^ was carried out. To separate CobB and OPRTase proteins, reactions were loaded onto an anionic exchange column (Hi‐trap Q HP; GE Healthcare). The column was previously equilibrated with conservation buffer and proteins were separated using a linear gradient of NaCl from 0.05 to 1 m at a flow rate of 5 mL·min^−1^.

### Enzymatic assay

Orotate phosphoribosyltransferase activity was assayed as previously described [81]. The assays were performed in conservation buffer, with the addition of 5 mm MgCl_2_, 0.2 mm orotate and 1 mm PRPP for the forward reaction, and 3 mm MgCl_2_, 2 mm of PPi and 0.1 mm of OMP for the reverse reaction. The reactions were carried out at 30 °C in a total volume of 100 μL and started by the addition of OPRTase. The assay was monitored by the decrease in absorbance at 295 nm caused by the conversion of orotate in OMP in the forward reaction or by the increase in absorbance at 295 nm caused by orotate formation in the reverse reaction. Specific activity was calculated using the extinction coefficient orotate at 295 nm (3950 m ^−1^·cm^−1^). One unit of activity was defined as the amount of enzyme needed to convert 1 μmol of orotate to OMP per minute (forward reaction) or 1 μmol of OMP to orotate per minute (reverse reaction), both at 30 °C. Reactions were carried out in triplicate. Orotate concentration was varied from 0.5 to 200 μm at 1 mm PRPP, PRPP concentration was varied from 12.5 to 1000 μm at 0.2 mm orotate, PPi concentration ranged from 0.005 to 2 mm at 0.1 mm OMP, and OMP was varied from 0.001 to 0.1 mm at 2 mm PPi. Kinetic parameters were determined employing prism v7 (GraphPad, San Diego, CA, USA) analytical software and standard deviations were determined from the replicates.

Aspartate carbamoyltransferase activity was assayed as previously described [82]. To study the thermostability of OPRTase, ATCase and acetylated and deacetylated proteins, aliquots of the proteins were subjected to different temperatures for a fixed period of time, and then the activity of the enzymes was measured.

### Extracellular metabolite quantification and physiological study

*Escherichia coli* K12 wt, *E. coli* K12 Δ*cobB* and *E. coli* K12 Δ*pyrE* transformed with pSF‐pMB1′‐BAD‐*mbp‐pyrE*, pRSF‐BAD‐*mbp*‐*pyrE* ^26AcK^ or pRSF‐BAD‐*mbp*‐*pyrE* ^103AcK^ vectors were grown in TB7 medium supplemented with glycerol 40 mm. Glycerol was used for the correct protein expression under the control of the P_BAD_ promoter. Cultures were induced with arabinose 2 mm at 0.3 OD_600_, and for acetylated protein expression, nicotinamide 20 mm and *N*ε‐acetyl‐l‐lysine 10 mm were added before induction. Kinetic and stoichiometric parameters were calculated, and a specific growth rate was determined [83]. To quantify glycerol consumption and extracellular orotate concentration 1 mL of culture samples at different growth times were taken. Pellets were harvested by centrifugation at 4 °C for 1 min at 12 000 r.p.m. and discarded, and supernatants were frozen until analysis. Extracellular metabolites and glycerol consumption were analysed by HPLC using an ion exclusion column (ICSep Coregel 87H3; Transgenomic, Omana, NE, USA) and equipped with UV and refractive index detectors (Shimadzu Scientific Instruments, Kyoto, Japan). The mobile phase was 5 mm H_2_SO_4_ flowing at 1 mL·min^−1^ and 65 °C.

### LC–MS/MS protein acetylation identification

To evaluate the acetylation level of proteins LC–MS/MS assay was carried out. Proteins were alkylated by incubation with 100 mm iodoacetamide for 30 min in the dark at room temperature. Samples were digested with 1 μg Trypsin Gold (Promega, Madison, WI, USA) (1 : 100 w/w) for 3 h at 37 °C, and the reaction was stopped by the addition of 0.1% formic acid. Tryptic peptides were dried employing a vacuum evaporator and then were separated and analysed by LC–MS.

The separation and analysis of the tryptic digests of the samples were performed with an HPLC/MS system consisting of an Agilent 1290 Infinity II Series HPLC (Agilent Technologies) connected to an Agilent 6550 Q‐TOF Mass Spectrometer (Agilent Technologies) using an Agilent Jet Stream Dual electrospray (AJS‐Dual ESI) interface. Dry samples from trypsin digestion were resuspended in 20 μL of buffer A, consisting of water/acetonitrile/formic acid (94.9 : 5 : 0.1). The sample was injected onto an Agilent AdvanceBio Peptide Mapping HPLC column (2.7 μm, 100 × 2.1 mm; Agilent Technologies), thermostatted at 50 °C, at a flow rate of 0.4 mL·min^−1^. The digested peptides were eluted using a linear gradient 0–40% phase B (phase B: water/acetonitrile/formic acid, 10 : 89.9 : 0.1) for 40 min followed by a linear gradient of 40–95% phase B for 8 min. 95% B was maintained for 3 min. The mass spectrometer was operated in the positive mode. The nebulizer gas pressure was set to 35 psi, whereas the drying gas flow was set to 14 L·min^−1^ at a temperature of 300 °C, and the sheath gas flow was set to 11 L·min^−1^ at a temperature of 250 °C. The capillary spray, nozzle, fragmentor and octopole RF Vpp voltages were 3500, 100, 360 and 750 V, respectively. Profile data were acquired for both MS and MS/MS scans in extended dynamic range mode at 4 GHz. MS and MS/MS mass range were 50–1700 *m*/*z* and scan rates were 8 spectra·s^−1^ for MS and 3 spectra·s^−1^ for MS/MS. Auto MS/MS mode was used with precursor selection by abundance and a maximum of 20 precursors selected per cycle. A ramped collision energy was used with a slope of 3.68 and an offset of −4.28. The same ion was rejected after two consecutive spectra. Data processing and analysis were performed Spectrum Mill MS Proteomics Workbench (Rev B.06.00.201; Agilent Technologies).

### Western blot assay

To study protein lysine acetylation, samples were separated on 10% acrylamide SDS/PAGE and transferred to polyvinylidene fluoride (PVDF) membranes employing a semidry transfer unit (Trans‐Blot Turbo Transfer System; Bio‐Rad, Hercules, CA, USA). The membranes were incubated with a primary rabbit monoclonal anti‐acetyl Lysine antibody (anti‐AcK) (ImmuneChem, Burnaby, BC, Canada) and with an HRP‐conjugated goat anti‐rabbit secondary antibody (Santa Cruz Biotechnology, Dallas, TX, USA). Finally, the membrane was incubated with SuperSignal™ West Pico Chemiluminescent Substrate (Thermo Fisher Scientific) for 10 min and revealed with a chemiluminescence Amersham Imager 600 (GE Healthcare). To study the expression of OPRTase‐26AcK and OPRTase‐103AcK proteins under P_BAD_ promoter (Fig. 14A), the primary monoclonal mouse anti‐(His)_6_ antibody (Thermo Fisher Scientific) was used, and an HRP‐conjugated goat anti‐mouse antibody (Invitrogen, Waltham, MA, USA) was used as a secondary antibody.

### Differential scanning calorimetry

The Differential scanning calorimetry (DSC) technique was used for thermodynamic analysis of denaturation and stability of ATCase control, ATCase acetylated and ATCase deacetylated. A MicroCal PEAQ‐DSC microcalorimeter (Malvern Panalytical, Malvern, UK) was employed. Samples were run from 30 °C to 100 °C at a rate of 1.25 °C·min^−1^, and the buffer containing the proteins (150 mm Tris–HCl, 50 mm NaCl, pH 7.5) was used as a control. The values of the measured parameters were studied and relativised to the protein concentration using origin 7.0 software (OriginLab Corporation, Northampton, MA, USA).

## Conflict of interest

The authors declare no conflict of interest.

## Author contributions

GL‐T, JG‐J and TDP have planned experiments; GL‐T and JG‐J have performed experiments and analysed data; RAS‐M, AMV, AO and MCD have contributed reagents or other essential material; GL‐T wrote the paper; JG‐J, RAS‐M, MCD, AO and TDP are involved in writing—review and editing; TDP is involved in supervision, project administration and funding acquisition. All authors have read and agreed to the published version of the manuscript.

### Peer review

The peer review history for this article is available at https://publons.com/publon/10.1111/febs.16598.

## Supporting information


**Table S1.** Plasmids, strains and primers employed in the study.

## Data Availability

All data are included in the article and Supporting Information.
