# Supplementary material for: Regulation of the pyrimidine biosynthetic pathway by lysine acetylation of E. coli OPRTase
Source: FEBS J. 2022 Sep 2;290(2):442–64. doi: 10.1111/febs.16598 (PMC10087573; doi:10.1111/febs.16598)
Supplement: Supplementary file 1 — Table S1. Plasmids, strains and primers employed in the study. [file FEBS-290-442-s001.pdf]

Regulation of the pyrimidine biosynthetic pathway by lysine acetylation of *E. coli* OPRTase

Gema Lozano-Terol[†], Julia Gallego-Jara[†], Rosa Alba Sola-Martínez, Álvaro Ortega, Adrián Martínez Vivancos, Manuel Cánovas Díaz and Teresa de Diego Puente[*]

Department of Biochemistry and Molecular Biology and Immunology (B), Faculty of Chemistry, University of Murcia, Campus of Espinardo, Regional Campus of International Excellence "Campus Mare Nostrum", Murcia, Spain

[*]Correspondence:
Teresa de Diego Puente. Department of Biochemistry and Molecular Biology and Immunology (B), Faculty of Chemistry, University of Murcia, Campus of Espinardo, Regional Campus of International Excellence "Campus Mare Nostrum", Murcia, Spain
Tel: +34 868887397
Email: tdp@um.es

[†]These authors have contributed equally to this work

Table S1. Plasmids, strains and primers employed in study.

Plasmid	Description	Source
**pKD13**	Amp^R/Kam^R, resistance cassette flanked by FRT regions.	Yale *E. coli* Genetic Stock Center
**pKD46**	Amp^R, this plasmid is temperature sensitive replication at 30°C and arabinose inducible Red recombinase expression.	Yale *E. coli* Genetic Stock Center
**pRSET-A**	Amp^R. pBR322 ori, P_{T7} promoter.	Invitrogen
**pET28a-*mbp***	Kan^R. lacI P_{T7} promoter, Maltose Binding Protein (MBP) Phusion tag	Lab deposit
**pET28a-*mbp*-*cobB***	Kan^R. lacI P_{T7} promoter, Maltose Binding Protein (MBP) Phusion tag. Encodes *cobB*	Lab deposit
**pRSET-*pyrE***	Amp^R. P_{T7} promoter. Encodes *pyrE*	This study
**pRSET-*pyrBI***	Amp^R. P_{T7} promoter. Encodes *pyrBI*	Lab deposit
**pET28a-*mbp-pyrE***	Kan^R. lacI P_{T7} promoter, Maltose Binding Protein (MBP) Phusion tag. Encodes *pyrE*	This study
**pET28a-*mbp*-*pyrE*^{26amber}**	Kan^R. lacI P_{T7} promoter, Maltose Binding Protein (MBP) Phusion tag. Encodes *pyrE*^{K26}	This study
**pET28a-*mbp*-*pyrE*^{103amber}**	Kan^R. lacI P_{T7} promoter, Maltose Binding Protein (MBP) Phusion tag. Encodes *pyrE*^{K103}	This study
**pRSF-Duet-1-acetyllysyl-tRNA-synthetase AcKRS3/*Mb*tRNA$_{CUA}$**	Kan^R. P_{T7} promoter. Encodes acetyl-lysyl-tRNA-synthetase AcKRS3/*Mb*tRNA$_{CUA}$	[1]
**pRSF-*mbp*-*pyrE*^{26AcK}**	Kan^R. P_{T7} promoter. Coexpression vector. Maltose Binding Protein (MBP) Phusion tag. Encodes acetyl-lysyl-tRNA-synthetase AcKRS3/*Mb*tRNA$_{CUA}$ and *pyrE*^{K26}	This study
**pRSF-*mbp*-*pyrE*^{103AcK}**	Kan^R. P_{T7} promoter. Coexpression vector. Maltose Binding Protein (MBP) Phusion tag. Encodes acetyl-lysyl-tRNA-synthetase AcKRS3/*Mb*tRNA$_{CUA}$ and *pyrE*^{K103}	This study
**pSF-pMB1´-BAD-YFP**	Amp^R. P_{BAD} promoter. Encode Kringle YFP.	[2]
**pSF-pMB1´-BAD-*mbp-pyrE***	Amp^R. P_{BAD} promoter. Maltose Binding Protein (MBP) Phusion tag. Encode *pyrE.*	This study
**pRSF-BAD-*mbp*-*pyrE*^{26AcK}**	Kan^R. P_{BAD} promoter. Coexpression vector. Maltose Binding Protein (MBP) Phusion tag. Encodes acetyl-lysyl-tRNA-synthetase AcKRS3/*Mb*tRNA$_{CUA}$ and *pyrE*^{K26}	This study

**pRSF-BAD-*mbp*-*pyrE*^{103AcK}**	Kan^R. P_{BAD} promoter. Coexpression vector. Maltose Binding Protein (MBP) Phusion tag. Encodes acetyl-lysyl-tRNA-synthetase AcKRS3/*Mb*$tRNA_{CUA}$ and *pyrE*^{K103}	This study

***E. coli* Strain**	**Description**	**Source**
**Top10F´**	F'[lacI^q Tn10(tetR)] *mcrA* Δ(*mrr-hsdRMS-mcrBC*) ϕ80*lac*ZΔM15 Δ*lacX74 deoR nupG recA1 araD139* Δ(*ara-leu*)7697 *galU galK rpsL*(StrR) *endA1* λ-	Invitrogen
**BL21 (DE3)**	F– *ompT gal dcm lon hsdSB*(*rB- mB-*) λ(DE3).	Promega
**K12 BW25113**	*lacI*$^+$*rrnB*$_{T14}$ Δ*lacZ*$_{WJ16}$ *hsdR*514Δ*araBAD*$_{AH33}$ Δ*rhaBAD*$_{LD78}$ *rph-1* Δ*(araB–D)567* Δ*(rhaD–B)568* Δ*lacZ4787*(::*rrnB-3*) *hsdR514 rph-1*	Invitrogen
**BL21 (DE3) Δ*ackA***	F– *ompT gal dcm lon hsdSB*(*rB- mB-*) λ(DE3) *ackA*:Kan^R	[3]
**BL21 (DE3) Δ*pta***	F– *ompT gal dcm lon hsdSB*(*rB- mB-*) λ(DE3) *pta*:Kan^R	[3]
**BL21 (DE3) Δ*patZ***	F– *ompT gal dcm lon hsdSB*(*rB- mB-*) λ(DE3) *patZ*:Kan^R	[3]
**BL21 (DE3) Δ*cobB***	F– *ompT gal dcm lon hsdSB*(*rB- mB-*) λ(DE3) *cobB*:Kan^R	[3]
**BL21 (DE3) Δ*yiaC***	F– *ompT gal dcm lon hsdSB*(*rB- mB-*) λ(DE3) *yiaC*:Kan^R	This study
**K12 BW25113 Δ*pyrE***	*lacI*$^+$*rrnB*$_{T14}$ Δ*lacZ*$_{WJ16}$ *hsdR*514Δ*araBAD*$_{AH33}$ Δ*rhaBAD*$_{LD78}$ *rph-1* Δ*(araB–D)567* Δ*(rhaD–B)568* Δ*lacZ4787*(::*rrnB-3*) *hsdR514 rph-1 pyrE:Kan*	This study
**K12 BW25113 Δ*cobB***	*lacI*$^+$*rrnB*$_{T14}$ Δ*lacZ*$_{WJ16}$ *hsdR*514Δ*araBAD*$_{AH33}$ Δ*rhaBAD*$_{LD78}$ *rph-1* Δ*(araB–D)567* Δ*(rhaD–B)568* Δ*lacZ4787*(::*rrnB-3*) *hsdR514 rph-1 cobB:Kan*	Lab deposit

**Primer**	**Sequence**
**Primers used for knockout strains constructions**	
***pyrE* KO Fwd**	AGTCGCCTTTTTTTTGTCTGTAGAAAAGTAAGATGAGGAGCGAAGGCATGATTCCGGGGATCCGTCGACC
***pyrE* KO Rev**	CCGGATGACTTTTCATCCGGCGAGTTTCTTTAAACGCCAAACTCTTCGCGTGTAGGCTGGAGCTGCTTCG
***yiaC* KO Fwd**	GTGAATGATCATGTGGTTGGCTGCTGTTGCTATCCGGGAAATAAACCATGATTCCGGGGATCCGTCGACC
***yiaC* KO Rev**	GGAAAAATACAACGGTCCGGAACTGACACTTACAGCGTTTGAACCACCGGTGTAGGCTGGAGCTGCTTCG
***pyrE* check Fwd**	ATCCATTGTAGCGACGCAGAA
***pyrE* check Rev**	ATACCGCCCGACGGATGATT
***yiaC* check Fwd**	TTTGTCGGCACCACAATCT
***yiaC* check Rev**	GTGCTGACCAAAGATCTCCC
**K1**	CAGTCATAGCCGAATAGCCT

**K2**	CGGTGCCCTGAATGAACTGC
**Primers used for mutagenesis and overexpression**	
***pyrE* pRSET Fwd**	GGTGGTGAATTCATGAAACCATATCAGCGCCA
***pyrE* pRSET Rev**	GGTGGTAAGCTTTTAAACGCCAAACTCTTCGC
***pyrE* pET28a Fwd**	GGTGGTGAATTCATGAAACCATATCAGCGCCA
***pyrE* pET28a Rev**	GGTGGTAAGCTTTTAAACGCCAAACTCTTCGC
***pyrE*^{K26AMBER} pET28a Fwd**	GCGAGTTTACGCTGTAGTCCGGGCGCAAAAGC
***pyrE*^{K26AMBER} pET28a Rev**	GCTTTTGCGCCCGGACTACAGCGTAAACTCGC
***pyrE*^{K103AMBER} pET28a Fwd**	CTTTAACCGCAAAGAAGCATAGGACCACGGTGAAGGCGGC
***pyrE*^{K103AMBER} pET28a Rev**	GCCGCCTTCACCGTGGTCCTATGCTTCTTTGCGGTTAAAG
***pyrE*^{K26AMBER} pRSF Fwd**	GGTGGTCTGCAGATGAAAATCGAAGAAGGTAAACT
***pyrE*^{K26AMBER} pRSF Rev**	GGTGGTGGTACCTTAAACGCCAAACTCTTCGCG
***pyrE*^{K103AMBER} pRSF Fwd**	GGTGGTCTGCAGATGAAAATCGAAGAAGGTAAACT
***pyrE*^{K103AMBER} pRSF Rev**	GGTGGTGGTACCTTAAACGCCAAACTCTTCGCG
**pSF-BAD Fwd**	AGAAGTTGTCTCCTCCTGCA
**pSF-BAD Rev**	TGGTGGGTACCTCCTTTGAA
***pyrE* pSF-BAD Fwd**	TTCAAAGGAGGTACCCACCAATGGAACATCACCATCACCAT
***pyrE* pSF-BAD Rev**	TGCAGGAGGAGACAACTTCTTTAAACGCCAAACTCTTCGC
**P_{BAD} *araC* Fwd**	ACTTACATTAATTGCGTTGCGTTATGACAACTTGACGGCTAC
**P_{BAD} *araC* Rev**	TAAAGTTAAACAAAATTATTTCTACTATGGAGAAACAGTAGAGAGTT
***pRSF-pyrE*$^{26AcK/103AcK}$ Fwd**	GTAGAAATAATTTTGTTTAACTTTA
***pRSF-pyrE*$^{26AcK/103AcK}$ Rev**	CGCAACGCAATTAATGTAAGT

1 de Boor S, Knyphausen P, Kuhlmann N, Wroblowski S, Brenig J, Scislowski L, Baldus L, Nolte H, Krüger M & Lammers M (2015) Small GTP-binding protein Ran is regulated by posttranslational lysine acetylation. *Proc Natl Acad Sci* **112**, 3679–3688.

2 Lozano Terol G, Gallego-jara J, Sola Martinez RA, Martinez Vivancos A, Canovas Diaz M & De Diego Puente T (2021) Impact of the Expression System on Recombinant Protein Production in *Escherichia coli* BL21. *Front Microbiol* **12**, 1–12.

3 Lozano Terol G, Gallego-Jara J, Sola Martínez RA, Cánovas Díaz M & De Diego Puente T (2019) Engineering protein production by rationally choosing a carbon and nitrogen source using *E. coli* BL21 acetate metabolism knockout strains. *Microb Cell Fact* **18**, 1–19.
